# Alarming and Calming: Opposing Roles of S100A8/S100A9 Dimers and Tetramers on Monocytes

**DOI:** 10.1002/advs.202201505

**Published:** 2022-10-30

**Authors:** Antonella Russo, Hendrik Schürmann, Matthias Brandt, Katja Scholz, Anna Livia L. Matos, David Grill, Julian Revenstorff, Maximilian Rembrink, Meike von Wulffen, Lena Fischer‐Riepe, Peter J. Hanley, Hans Häcker, Monika Prünster, Francisco Sánchez‐Madrid, Sven Hermann, Luisa Klotz, Volker Gerke, Timo Betz, Thomas Vogl, Johannes Roth

**Affiliations:** ^1^ Institute of Immunology University of Münster 48149 Münster Germany; ^2^ Cells in Motion Interfaculty Centre University of Münster 48149 Münster Germany; ^3^ Institute of Cell Biology Centre for Molecular Biology of Inflammation ZMBE University of Münster 48149 Münster Germany; ^4^ Institute of Medical Biochemistry Centre of Molecular Biology of Inflammation ZMBE University of Münster 48149 Münster Germany; ^5^ Faculty of Medicine HMU Health and Medical University Potsdam 14471 Potsdam Germany; ^6^ Department of Pathology Division of Microbiology and Immunology University of Utah Salt Lake City UT 84112 USA; ^7^ BioMedical Center Walter‐Brendel‐Centre for Experimental Medicine Ludwig‐Maximilians‐University Planegg‐Martinsried 82152 Munich Germany; ^8^ Immunology Service Hospital de la Princesa Universidad Autónoma de Madrid Instituto Investigación Sanitaria Princesa Madrid 28006 Spain; ^9^ Department of Vascular Biology and Inflammation Centro Nacional de Investigaciones Cardiovasculares (CNIC) Madrid 28029 Spain; ^10^ European Institute for Molecular Imaging (EIMI) University of Münster 48149 Münster Germany; ^11^ Department of Neurology with Institute of Translational Neurology University Hospital Muenster 48149 Muenster Germany; ^12^ Third Institute of Physics– Biophysics Georg August University Göttingen 37077 Göttingen Germany

**Keywords:** alarmin, calprotectin, CD69, MRP8/MRP14, migration, S100A8/S100A9 tetramer

## Abstract

Mechanisms keeping leukocytes distant of local inflammatory processes in a resting state despite systemic release of inflammatory triggers are a pivotal requirement for avoidance of overwhelming inflammation but are ill defined. Dimers of the alarmin S100A8/S100A9 activate Toll‐like receptor‐4 (TLR4) but extracellular calcium concentrations induce S100A8/S100A9‐tetramers preventing TLR4‐binding and limiting their inflammatory activity. So far, only antimicrobial functions of released S100A8/S100A9‐tetramers (calprotectin) are described. It is demonstrated that extracellular S100A8/S100A9 tetramers significantly dampen monocyte dynamics as adhesion, migration, and traction force generation in vitro and immigration of monocytes in a cutaneous granuloma model and inflammatory activity in a model of irritant contact dermatitis in vivo. Interestingly, these effects are not mediated by the well‐known binding of S100A8/S100A9‐dimers to TLR‐4 but specifically mediated by S100A8/S100A9‐tetramer interaction with CD69. Thus, the quaternary structure of these S100‐proteins determines distinct and even antagonistic effects mediated by different receptors. As S100A8/S100A9 are released primarily as dimers and subsequently associate to tetramers in the high extracellular calcium milieu, the same molecules promote inflammation locally (S100‐dimer/TLR4) but simultaneously protect the wider environment from overwhelming inflammation (S100‐tetramer/CD69).

## Graphical abstract

Mechanisms restricting local inflammation and preventing systemic spreading of diseases are hardly understood. Release of S100A8/S100A9‐dimers at local sites of inflammation leads to monocyte activation via TLR4 while calcium‐induced S100A8/S100A9‐tetramer formation in the extracellular space mitigates monocyte dynamics via CD69. Thus, different quaternary structures of the same molecules promote inflammation locally (S100‐dimer/TLR4) but simultaneously protect systemic spreading of inflammation (S100‐tetramer/CD69).

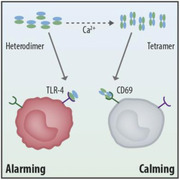

## 1 Introduction

Many molecular pathways activating inflammatory mechanisms or promoting cellular dynamics, migration, and recruitment of leukocytes during inflammation have been characterized during the last years. Pathways avoiding spreading of inflammatory activity and keeping leukocytes distant from the primary site of inflammation in a resting state; however, are less understood. The latter, however, is a pivotal requirement to avoid uncontrolled systemic spreading of inflammatory activity and to restrict inflammatory processes to local sites of infection or injury.

Monocytes and macrophages are major effector cells in almost every inflammatory disease. They show pro‐ as well as anti‐inflammatory properties depending on their stage of activation or differentiation. Regulated recruitment and differentiation of monocytes and macrophages are therefore crucial processes for physiological inflammatory responses.^[^ ^1^, ^2^ ^]^ However, we are only beginning to understand the mechanisms driving recruitment of specific populations such as monocytes/macrophages to sites of inflammation while simultaneously preventing systemic spreading of inflammation.

S100A8 and S100A9, originally described as myeloid related protein (MRP) 8 and MRP14,^[^ ^3^ ^]^ are cytosolic calcium‐binding proteins highly expressed in phagocytes such as neutrophils and monocytes. Both proteins belong to the family of danger‐associated molecular pattern molecules (DAMPs) or alarmins and are secreted as S100A8/S100A9‐heterodimers during the activation of phagocytes by an unconventional mechanism.^[^ ^4^, ^5^ ^]^ Heterodimer‐complexes promote inflammatory responses via Toll‐like receptor 4 (TLR4) signaling cascades.^[^ ^6^, ^7^, ^8^ ^]^ In many clinically relevant disorders, such as rheumatoid arthritis, inflammatory bowel diseases or psoriasis, S100A8/S100A9‐complexes are the most abundant alarmins expressed and released at local sites of inflammation, and the S100A8/S100A9‐complex is widely used as a biomarker under the brand name “calprotectin” for monitoring inflammatory disease activity.^[^ ^9^, ^10^, ^11^ ^]^ In the presence of extracellular calcium concentrations as found in the systemic circulation in vivo (≈2.09–2.54 mmol L^−1^) or under cell culture conditions in vitro (≈ 0.89 mmol L^−1^), two S100A8/S100A9‐dimers form rapidly a S100A8/S100A9‐tetramer.^[^ ^5^, ^8^, ^12^, ^13^ ^]^ S100‐tetramer formation results in steric shielding of the S100 amino acids responsible for the interaction with TLR4 in the interface of the tetramer; thus, preventing TLR4 binding of these S100‐proteins.^[^ ^8^ ^]^ Intracellular tetramer formation has been shown to modulate microtubule stability but the only function of extracellular S100A8/S100A9‐tetramers described so far was an antimicrobial activity by chelating bivalent cations as calcium, zinc, manganese or nickel, which are important for the growth of many pathogens but a functional relevance of the high systemic levels of this protein complex under noninfectious conditions has not been described so far.^[^ ^5^, ^14^, ^15^, ^16^ ^]^

Here, we demonstrate that monocytes lacking S100A8/S100A9 exhibit major alterations in their cell dynamics. Interestingly, these effects are reversed upon addition of extracellular S100A8/S100A9‐tetramers but not S100A8/S100A9‐dimers. We show that these phenotypes are not induced by S100A8/S100A9 binding to TLR4 but are specifically mediated by S100‐tetramers in a CD69‐dependent manner. The in vivo relevance of our novel findings could be verified by a reduced immigration of monocytes in a cutaneous granuloma model and diminished inflammation during irritant contact dermatitis in the presence of S100A8/S100A9‐tetramers. Thus, we can finally demonstrate different functions of dimeric and tetrameric S100A8/S100A9 complexes which either trigger local inflammatory activity via TLR4 (S100‐dimers) or systemically dampen migratory cell dynamics of monocytes via CD69 (S100‐tetramers) depending on their quaternary structure.

## 2 Results

### 2.1 Monocyte Migration and Morphology are Dependent on S100A8/S100A9 Expression

**Figure 1:**
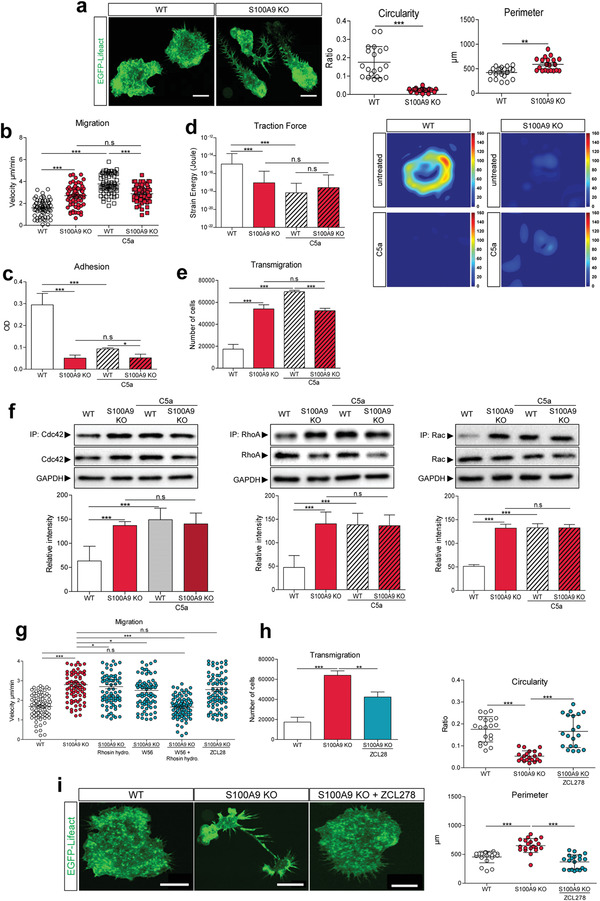
S100A8/S100A9 deficiency alters morphology and migration capacity of monocytes and depends on small GTPase activities. a) Imaging of Lifeact WT (*n* = 20) and Lifeact S100A9 KO monocytes (*n* = 20). The circularity and perimeter of these two cell types were determined by ImageJ. One‐way ANOVA with Bonferroni post‐test analysis. Scale bar = 10 µm. b) 2D migration assay experiment showing the speed of Lifeact WT (*n* = 66) and Lifeact S100A9 KO monocytes (*n* = 66) in a chemokine free and in a complement C5a gradient (20 nm) after 6 h of migration. One‐way ANOVA with Bonferroni post‐test analysis. c) Analysis of adhesion properties of untreated and 5 min complement C5a (20 nm) stimulated Lifeact WT and Lifeact S100A9 KO monocytes after 30 min of adhesion on 50 µg mL^−1^ fibronectin coated 96 well plate. Quantification was obtained via optical density of crystal violet stained adherent cells. One‐way ANOVA with Bonferroni post‐test analysis. d) Left panel, strain energy of Lifeact WT (*n* = 16) and Lifeact S100A9 KO monocytes (*n* = 16) under steady state conditions and after stimulation with complement C5a (20 nm). Mann–Whitney U test. Right panel shows representative force maps of untreated and complement C5a (20 nm) stimulated Lifeact WT and Lifeact S100A9 KO monocytes. e) Analysis of transmigrated Lifeact WT and Lifeact S100A9 KO monocytes in a chemokine free and chemokine rich environment (20 nm complement C5a) via a boyden chamber assay. f) Immunoprecipitation assays of Cdc42, RhoA, and Rac. Lifeact WT and Lifeact S100A9 KO monocytes were left untreated or stimulated for 5 min with the complement C5a (20 nm). Immunoprecipitated GTPases (GTP‐bound) and total amounts were analyzed by western blot. The intensity of the IP‐bands was measured via ImageJ. g,) 2D migration assay experiment showing the speed of Lifeact WT (*n* = 82), Lifeact S100A9 KO monocytes (*n* = 82), and Lifeact S100A9 KO monocytes treated for 30 min with Rhosin hydrochloride (30 µm; 82 cells), W56 (250 µm; 82 cells), Rhosin hydrochloride and W56 (82 cells), and ZCL28 (50 µm; 82 cells) after 6 h of migration. One‐way ANOVA with Bonferroni post‐test analysis. h) Transmigration of Lifeact WT, Lifeact S100A9 KO, and Lifeact S100A9 KO monocytes treated with ZCL278 (30 min, 50 µm) in a chemokine free set‐up, via a boyden chamber assay. One‐way ANOVA with Bonferroni post‐test analysis. i) Imaging of Lifeact WT (*n* = 20), Lifeact S100A9 KO (*n* = 20), and Lifeact S100A9 KO monocytes treated for 30 min with ZCL278 (50 µm) (*n* = 20, Scale bar = 10 µm). Morphological (circularity and perimeter) analysis was performed using ImageJ. Data were pooled from three independent experiments. For statistical analysis, one‐way ANOVA with Bonferroni post‐test analysis was used for all experiments. n.s: not significant, * *P* < 0.05, ** *P* < 0.01, *** *P* < 0.001.

Using the ER‐Hoxb8 system,^[^ ^17^ ^]^ we generated transiently immortalized myeloid progenitor cells of enhanced green fluorescent protein (EGFP)‐Lifeact wild type (WT) and EGFP‐Lifeact mice crossed with S100A9 knock‐out (KO) mice which were subsequently differentiated to monocytes (day 3). KO of S100A9 in mice is generally associated with absence of both S100A9 and S100A8 at the protein level, resulting functionally in S100A8/S100A9 double KO mice and cells.^[^ ^18^, ^19^ ^]^ We observed significant morphological differences between ER‐Hoxb8 WT and S100A9 KO. While S100A9 KO cells exhibited elongated trailing ends (uropods) with numerous filopodia‐like extensions, which are probably largely retraction fibers, WT cells had compact morphologies. This altered morphology was confirmed by quantifying the circularity as well as the perimeter of S100A9 KO and WT cells (**Figure** **1**a).

To ensure that these cellular differences are not caused by the artificial ER‐Hoxb8 expression, we also compared primary bone marrow derived cells (BMC) obtained from WT and S100A9 knockout mice. BMCs were differentiated to macrophages for 7 days in the presence of macrophage colony‐stimulating factor (M‐CSF) and analyzed over time regarding the expression of surface markers such as Cd11b, Ly6c and F4/80 as well as expression of S100A9 (Figure S1a, Supporting Information). Bone marrow derived WT and S100A9 KO monocytes (d3) showed major differences in their morphology comparable to the results observed in the ER‐Hoxb8 system (Figure S1b, Supporting Information; Figure 1a). We next analyzed the migratory behavior. In the absence of a chemokine gradient, S100A9 KO monocytes migrated with a higher speed compared to WT monocytes. In the presence of the chemoattractant complement C5a; however, WT monocytes increased their migratory speed toward the chemotactic stimulus whereas S100A9 KO cells did not show any additional speed increase (Figure 1b) and migrated slower than the WT cells under this condition. Furthermore, under steady state conditions, WT monocytes showed stronger adherence to fibronectin compared to S100A9 KO cells. In WT cells, this strong adhesion was lost after complement C5a stimulation whereas S100A9 KO cells did not change their already low basal adhesion properties (Figure 1c). To analyze whether the adhesion differences reflect the traction force generation capacities, we studied the forces of these cells transferred to extracellular matrix structures using traction force microscopy.^[^ ^20^ ^]^ EGFP‐Lifeact WT and EGFP‐Lifeact S100A9 KO monocytes were seeded on fibronectin coated polyacrylamide gels (Young's modulus: 3 kPa) containing red fluorescent beads. Analyses of the strain energy showed a significantly higher deformation of the gel; and hence, larger force generation by unstimulated WT compared to S100A9 KO monocytes. After activation of WT cells with complement C5a, the deformation was significantly reduced. In contrast, the strain energy of unstimulated S100A9 KO monocytes was already low in the steady state condition and did not further decrease after complement C5a stimulation (Figure 1d). In order to assess the transmigration ability of WT and S100A9 KO monocytes, we performed two‐chamber transmigration assays. Consistent with the observed elevated chemotactic migration speed, significantly more S100A9 KO monocytes transmigrated compared to WT cells. Chemoattractant complement C5a stimulation had no effect on the transmigration of S100A9 KO cells but significantly enhanced the transmigration of WT cells (Figure 1e). To rule out that S100A9 KO monocytes are generally not responsive to complement C5a, we used a fluorescent calcium indicator to monitor calcium signaling. No differences in complement C5a‐induced calcium signaling could be observed between WT and S100A9 KO monocytes (Figure S2, Supporting Information).

Hence, regarding morphology, migration, and force generation, the S100A9 KO cells resemble the activated WT, suggesting that S1008/S100A9 is required to restrain monocyte activation.

### 2.2 Increased Activities of Small Rho GTPases Alter Cell Shape and Migration Speed in S100A9 KO Cells

To gain insight into the mechanisms leading to the observed activation like phenotype in the S100A9 KO cells, we investigated intracellular signaling pathways relevant for migration and force generation. Small GTPases of the Rho family are regulators of the cytoskeleton, membrane dynamics, and cell shape, switching from an inactive GDP‐ to an active GTP‐bound form via guanine nucleotide exchange factors (GEFs) and vice versa from a GTP‐ to GDP‐bound inactive form via GTPase‐activating proteins (GAPs).^[^ ^21^ ^]^ S100A9 KO cells expressed slightly elevated levels of total Cdc42. To study more relevant effects on Rho GTPase activation, we quantified the GTP‐bound form of Cdc42, RhoA, and Rac by immunoprecipitation assays. Under steady state conditions, S100A9 KO cells expressed significantly higher amounts of active GTP‐bound Cdc42 even after normalization to the slightly elevated total Cdc42 levels. The same was true for RhoA and Rac compared to WT monocytes. Activation of all GTPases was not further enhanced after stimulation with complement C5a, in contrast to WT monocytes, which responded to complement C5a stimulation with significantly increased GTP‐bound Rho GTPase levels (Figure 1f).

Incubation of S100A9 KO monocytes with specific GTPase inhibitors (ZCL28 for Cdc42; Rhosin hydrochloride for RhoA; and W56 for Rac) for 30 min reduced the GTP‐bound amounts of Cdc42, RhoA, and Rac to those present in WT cells, confirming the efficacy of these inhibitors in our experimental settings (Figure S3a, Supporting Information).

Consistent with its importance in cell migration, specific inhibition of either RhoA or Rac slightly reduced the migration speed of S100A9 KO cells, whereas simultaneous inhibition of both RhoA and Rac reduced their migratory speed to the WT level, while Cdc42 inhibition had no effect on velocity (Figure 1g). In contrast to the effects seen on velocity, inhibition of Cdc42 significantly reduced the number of transmigrating S100A9 KO monocytes (Figure 1h). Inhibition of RhoA or Rac, as well as simultaneous inhibition of both Rho GTPases, had no effect on the transmigration rates (Figure S3b, Supporting Information). We next analyzed whether the morphological differences seen in S100A9 KO cells are also dependent of GTPase activities. According to the well‐known fact that Cdc42 controls filopodia formation via formins, inhibition of Cdc42 converted the morphology of S100A9 KO to that seen in WT cells, inducing rounded‐up morphologies with only few filopodia‐like structures (Figure 1i). Our data demonstrate that lack of S100A8/S100A9 changes the activity of Rho GTPases and that the activity of Cdc42 is the dominant factor responsible for changes in directed transmigration properties and dysregulation of monocyte morphology in S100A9 KO cells under steady state conditions. On the other hand, effects of S100A8/S100A9 on RhoA and Rac activities are the molecular basis for changes in the migratory speed in S100A9 KO cells.

### 2.3 STAT3 is a Central Signaling Molecule Affected by S100A8/S100A9

**Figure 2:**
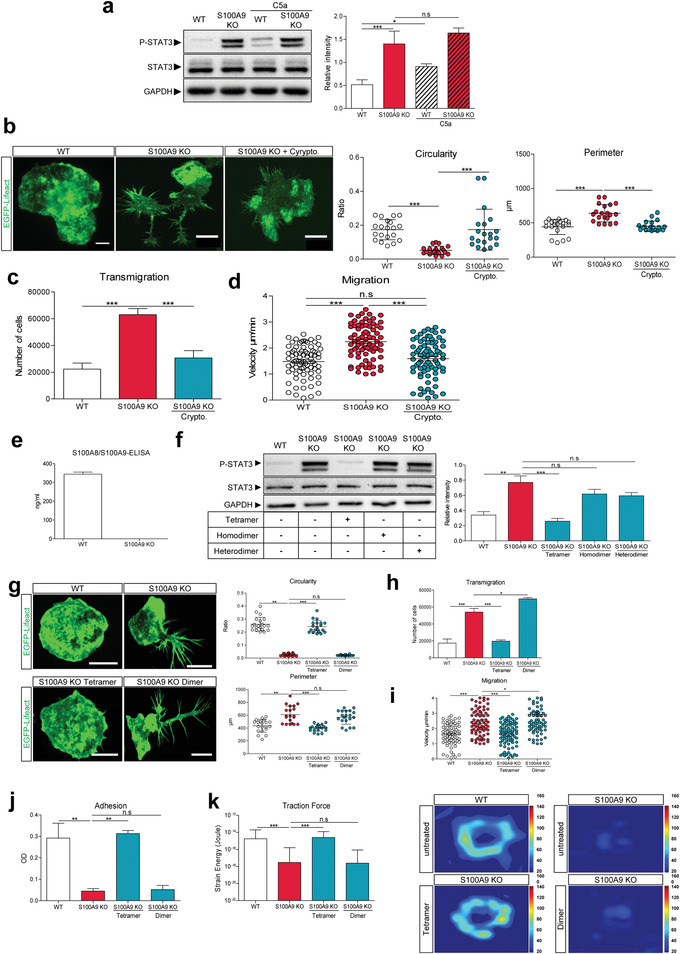
Increased P‐STAT3 activity in S100A8/S100A9 deficient EGFP‐Lifeact ER‐Hoxb8 monocytes and extracellular S100A8/S100A9‐tetramers rescues the S100A9 KO to WT monocytes. a) Immunoblot analysis of P‐STAT3 and total STAT3 of Lifeact WT and Lifeact S100A9 KO monocytes in the presence and absence of complement C5a (20 nm, 5 min). Quantification of STAT3 activation corresponds to the ratio of P‐STAT3 on total STAT3 levels after normalization to their GAPDH bands. b) Imaging of Lifeact WT (*n* = 20), Lifeact S100A9 KO (*n* = 20), and Lifeact S100A9 KO monocytes treated for 30 min with 5 µm of Crypto. (*n* = 20). Circularity and perimeter were analyzed via ImageJ software. Scale bar = 10 µm. c) Transmigration of Lifeact WT, Lifeact S100A9 KO, as well as Lifeact S100A9 KO monocytes treated for 30 min with Crypto (5 µm) was analyzed in a chemokine free environment. d) 2D migration assay showing the speed of Lifeact WT (*n* = 77), Lifeact S100A9 KO (*n* = 77), and Lifeact S100A9 KO monocytes treated for 30 min with Crypto (5 µm; *n* = 77) in the absence of a chemokine gradient after 6 h of migration. e) S100A8/S100A9 enzyme‐linked immunosorbent assay (ELISA) of the supernatant of untreated Lifeact WT and Lifeact S100A9 KO monocytes. f) Immunoblot analysis of P‐STAT3 and STAT3 in Lifeact WT and Lifeact S100A9 KO monocytes as well as Lifeact S100A9 KO cells treated with S100A8/S100A9‐tetramer (250 ng mL^−1^), S100A8/S100A8‐homodimer (250 ng mL^−1^), or S100A8/S100A9‐N70A‐heterodimer (250 ng mL^−1^) for 3 days of differentiation. g) Imaging of Lifeact WT (*n* = 20), Lifeact S100A9 KO (*n* = 20), and Lifeact S100A9 KO monocytes treated with S100A8/S100A9‐tetramer (250 ng mL^−1^; *n* = 20) or S100A8/S100A8‐homodimer (250 ng mL^−1^; *n* = 20) for 3 days. Scale bar = 10 µm. Circularity and perimeter were analyzed via ImageJ software. h) Transmigration rates of Lifeact WT, Lifeact S100A9 KO, and Lifeact S100A9 KO monocytes treated with S100A8/S100A9‐tetramer (250 ng mL^−1^) or S100A8/S100A8‐homodimer (250 ng mL^−1^) were analyzed via a boyden chamber system in a chemokine free condition. i) 2D migration experiment showing the speed of Lifeact WT (*n* = 77), Lifeact S100A9 KO (*n* = 77), and Lifeact S100A9 KO monocytes treated for 3 days with S100A8/S100A9‐tetramer (250 ng mL^−1^; *n* = 77) or S100A8/S100A8‐homodimer (250 ng mL^−1^; *n* = 77) after 6 h of migration. j) Adhesion properties of Lifeact WT, Lifeact S100A9 KO monocytes, and Lifeact S100A9 KO cells treated with S100A8/S100A9‐tetramer (250 ng mL^−1^) or S100A8/S100A8‐homodimer (250 ng mL^−1^). K) Left panel, traction force microscopy of Lifeact WT (*n* = 16), Lifeact S100A9 KO monocytes (*n* = 16), and Lifeact S100A9 KO monocytes (*n* = 16) treated for 3 days with S100A8/S100A9‐tetramer (250 ng mL^−1^; *n* = 19) or S100A8/S100A8‐homodimer (250 ng mL^−1^; *n* = 15). Right panel shows the representative force maps. Data were pooled from three independent experiments. For statistical analysis, one‐way ANOVA with Bonferroni post‐test analysis was used for all experiments. n.s: not significant, * *P* < 0.05, ** *P* < 0.01, and *** *P* < 0.001.

Previous work has shown that signal transducer and activator of transcription 3 (STAT3) is involved in cell migration and actin cytoskeleton reorganization.^[^ ^22^, ^23^ ^]^ We analyzed the total levels of STAT1, 3, 5, and 6 and the phosphorylation status of all four STAT (P‐STAT) proteins. While all four STAT proteins were equally expressed in WT‐ and S100A9 KO monocytes, only S100A9 KO monocytes had significantly higher levels of P‐STAT3 in the unstimulated or steady state condition compared to WT monocytes, and after stimulation with complement C5a, only WT cells responded with significantly increased phosphorylation of STAT3 (**Figure** **2**a). For P‐STAT1, 5, and 6, we could not detect any different bands in the western blot (Figure S4a, Supporting Information).

In accordance with the western blot results, S100A9 KO monocytes showed a significantly higher ratio of nuclear‐to‐cytoplasmic P‐STAT3 as compared to WT cells, which was reverted after Cryptotanshinone (STAT3 inhibitor) treatment (Figure S4 c,d, Supporting Information). Treatment with Cryptotanshinone also reduced the amount of active Cdc42, RhoA, and Rac in S100A9 KO monocytes to the levels found in WT cells (Figure S4d, Supporting Information). Moreover, blocking STAT3 phosphorylation restored morphology (Figure 2b) as well as transmigration rates (Figure 2c) and migration speeds (Figure 2d) of S100A9 KO cells to WT levels. Knock‐out of STAT3 alone did not alter wild‐type morphology and STAT3 KO on the S100A9 KO background even restored the wild‐type morphology. Only WT monocytes were able to respond to C5a (Figure S4e, Supporting Information).

### 2.4 Extracellular S100A8/S100A9‐Tetramers Control Cell Dynamics of Monocytes

Next, we investigated whether the observed effects on cell dynamics described above were due to intracellular S100A8/S100A9 or S100A8/S100A9 proteins that following secretion, could act in an autocrine manner. During differentiation of monocytes to macrophages, the cells constitutively released S100A8/S100A9‐dimers which rapidly formed S100A8/S100A9‐tetramers due to the high calcium ion concentrations present in the cell culture medium.^[^ ^8^ ^]^ We performed a S100A8/S100A9‐ELISA which detects both S100A8/S100A9‐dimers and S100A8/S100A9‐tetramers to quantify the amounts of spontaneous secretion of S100A8/S100A9 in unstimulated WT and S100A9KO monocytes. As expected, no S100A8/S100A9 could be detected in S100A9 KO monocytes, whereas for WT cells, we detected ≈350 ng mL^−1^ S100A8/S100A9 in the supernatant (Figure 2e). To analyze the effect of the extracellular alarmin, we added S100A8/S100A9‐tetramers, S100A8‐homodimers, and S100A8/S100A9‐N70A mutated heterodimers (deletion of the high affinity calcium‐binding site in S100A9) to S100A9 KO monocytes. S100A8‐homodimers and S100A8/S100A9‐N70A mutated heterodimers are unable to form tetramers and; are therefore, constitutively active triggers of TLR4.^[^ ^8^ ^]^ Interestingly, addition of wildtype S100A8/S100A9‐complexes, which are able to form tetramers, significantly reduced the phosphorylation state of STAT3 (Figure 2f) and its nuclear translocation (Figure S5a, Supporting Information) in S100A9 KO monocytes to a level similar to WT cells, whereas both forms of constitutively active dimers had no effect. For WT monocytes we could not observe additional effects after stimulation with tetramers or dimers (data not shown).

We therefore used S100A8‐homodimers and S100A8/S100A9‐tetramers in the following experiments because all S100‐dimers show a similar activation of TLR4^[^ ^8^ ^]^ and purification of mutated S100A8/S100A9‐N70A heterodimers needs a complex and time‐consuming de‐/renaturation protocol. The morphological changes of S100A9 KO monocytes were also completely reversed upon tetramer stimulation, while no effects could be observed for the pro‐inflammatory active homodimer (Figure 2g). Moreover, in tetramer‐stimulated S100A9 KO cells, the amount of active GTP‐bound Rho‐GTPases was reduced to the level of WT cells (Figure S5b, Supporting Information). Next, we investigated the cellular dynamics of S100A9 KO cells treated with S100‐dimers or S100‐tetramers using transmigration assays (Figure 2h), 2D migration (Figure 2i), and adhesion assays (Figure 2j) as well as traction force microscopy (Figure 2k). S100A9 KO monocytes behaved similar to unstimulated WT monocytes after treatment with S100‐tetramers but showed no changes after application of S100‐dimers.

### 2.5 Extracellular S100A8/S100A9‐Tetramers Control STAT3 Activation Via Expression of SOCS3

**Figure 3:**
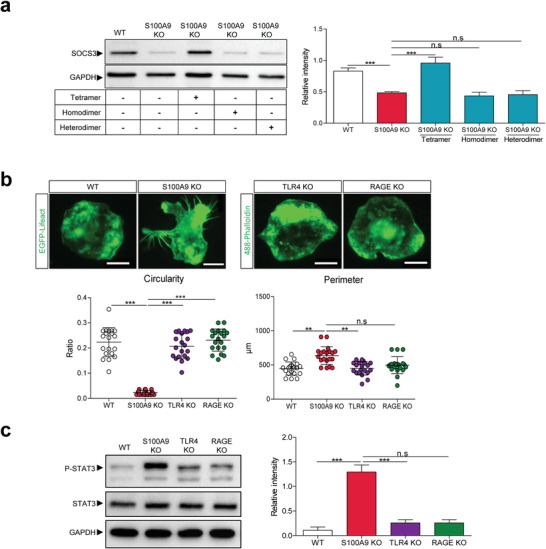
TLR4 and RAGE are not involved in S100‐tetramer specific effects on monocytes. a) Immunoblot analysis of SOCS3 in Lifeact WT, Lifeact S100A9 KO, as well as Lifeact S100A9 KO monocytes stimulated with either S100A8/S100A9‐tetramer (250 ng mL^−1^) or S100A8/S100A8‐homodimer (250 ng mL^−1^) or S100A8/S100A9‐heterodimer (250 ng mL^−1^). b) Morphological analysis of Lifeact WT (*n* = 20) and Lifeact S100A9 KO (*n* = 20) and Alexa Fluor 488‐phalloidin labeled TLR‐4 KO (*n* = 20) and RAGE KO monocytes (*n* = 20). Scale bar = 10 µm. c) Immunoblot analysis of P‐STAT3 and total STAT3 in Lifeact WT and Lifeact S100A9 KO as well as TLR4 KO and RAGE KO monocytes. Data are pooled from three independent experiments. For statistical analysis, one‐way ANOVA with Bonferroni post‐test analysis was used. n.s: not significant, ** *P* < 0.01, and *** *P* < 0.001.

The family of suppressor of cytokine signaling (SOCS) proteins are feedback inhibitors of the Janus kinase (JAK) and signal transducer and activator of transcription (STAT) signaling pathway; especially SOCS3 is a known inhibitor of STAT3 phosphorylation.^[^ ^24^ ^]^ We analyzed SOCS3 expression via western blot and observed higher SOCS3 levels in WT compared to S100A9 KO monocytes. Again, S100‐tetramer stimulation significantly increased SOCS3 expression in S100A9 KO monocytes, while S100‐dimers had no effect (**Figure** **3**a).

We next addressed the question of which receptor may mediate the effects of S100‐tetramers on cell dynamics in monocytes. First, we compared the morphology of ER‐Hoxb8 monocytes lacking two well studied S100A8/S100A9‐receptors, TLR4 or receptor for advanced glycation end products (RAGE), with WT controls. As clearly shown in Figure 3b, TLR4 KO as well as RAGE KO monocytes showed the same morphology, circularity, and perimeter as WT cells. In addition, the amount of P‐STAT3 in TLR4 KO and RAGE KO monocytes was similar to WT monocytes and significantly lower compared to S100A9 KOs (Figure 3c). Accordingly, only the S100A9 KO monocytes presented higher P‐STAT3 intensity in the nucleus compared to WT, TLR4 KO, and RAGE KO monocytes, as indicated by immunofluorescence staining (Figure S6, Supporting Information).

These data exclude the involvement of TLR4 and RAGE in the effects described above and implicate the involvement of another receptor in this new mechanism, which is in accordance with the fact that S100‐dimers do not correct the S100A9 KO phenotype.

### 2.6 CD69 is a Specific Receptor for S100A8/S100A9 Tetramers on Monocytes

**Figure 4:**
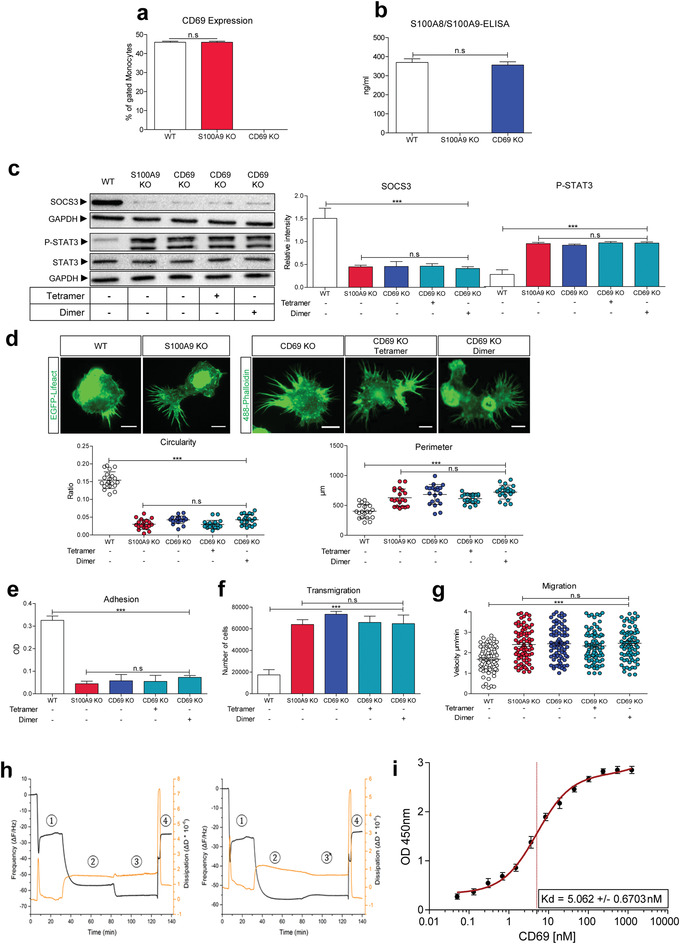
S100A8/S100A9‐tetramers signal via the early activation marker CD69. a) Fluorescence activated cell sorting (FACS) analysis of CD69 surface expression in Lifeact WT, Lifeact S100A9 KO, and CD69 KO monocytes. b) S100A8/S100A9 ELISA of Lifeact WT, Lifeact S100A9 KO, and CD69 KO monocytes supernatant. c) Immunoblot of SOCS3 in the upper panel and P‐STAT3/STAT3 in the lower panel. Lifeact WT, Lifeact S100A9 KO, CD69 KO, and CD69 KO monocytes treated with S100A8/S100A9‐tetramer (250 ng mL^−1^) or S100A8/S100A8‐homodimer (250 ng mL^−1^). d) Morphological changes in circularity and perimeter of untreated Lifeact WT (*n* = 20), Lifeact S100A9 KO (*n* = 20), CD69 KO, and CD69 KO monocytes treated with S100A8/S100A9‐tetramer (250 ng mL^−1^; *n* = 20) or S100A8/S100A8‐homodimer (250 ng mL^−1^; *n* = 20). Scale bar = 10 µm. Circularity and perimeter were analyzed via ImageJ software. Untreated Lifeact WT, Lifeact S100A9 KO, CD69 KO, and CD69 KO monocytes treated with S100A8/S100A9‐tetramer (250 ng mL^−1^) or S100A8/S100A8‐homodimer (250 ng mL^−1^) were analyzed for: e) adhesion properties, f) transmigration abilities, g) 2D migration experiment showing the speed of Lifeact WT (*n* = 77), Lifeact S100A9 KO (*n* = 77), CD69 KO monocytes, and CD69 KO monocytes treated with S100A8/S100A9‐tetramer (*n* = 77) or S100A8/S100A8‐homodimer (*n* = 77) in the absence of a chemokine gradient after 6 h of migration. h) Quartz Crystal microbalance analysis, black lines show the resonance frequency, and the orange lines the dissipation shifts of the quartz sensors during measurement over time. 4h‐(1)) formation of the lipid bilayer. 4h‐(2)) CD69 receptor (250 nm) adsorption onto the lipid bilayer. 4h‐(3)) S100A8/S100A9‐tetramer (1 µg mL^−1^) adsorption adds mass without changing the dissipation, indicating that the lipid film is not perturbed (left graph). 4h‐(3)) S100A8/S100A9‐N70A‐heterodimer (1 µg mL^−1^) adsorption does not add mass (right graph). 4h‐(4)) The recovery of the frequency baseline upon Imidazole chelation indicates total desorption of the receptor from the lipid bilayer. i) Estimation of the binding constant of CD69 to S100‐tetramer. The constant was calculated using the equation of a 1:1 binding model as described by Eble.^[^ ^26^ ^]^ Each dot represents the mean value of three independent experiments. For statistical analysis, one‐way ANOVA with Bonferroni post‐test analysis was used for (a–g). n.s.: not significant, * *P* < 0.05, ** *P* < 0.01, *** *P* < 0.001. Significance on capped line is the same for all conditions referred to as the WT or S100A9 KO control, respectively.

CD69 is a so‐called early activation marker present on hematopoietic stem cells and many different immune cells and is known to regulate the phosphorylation of STAT3 via SOCS3.^[^ ^25^ ^]^ Therefore, CD69 constituted a candidate receptor for S100A8/S100A9 tetramers, in particular, because an interaction between S100A8/S100A9 and CD69 has been reported before in T cells.^[^ ^25^ ^]^ We first analyzed the expression of CD69 in WT, S100A9 KO, and in CD69 KO monocytes. No differences could be detected in the expression levels of CD69 between WT and S100A9 KO monocytes and as expected, lack of CD69 expression was confirmed in CD69 KO monocytes (**Figure** **4**a).

Next, we analyzed the spontaneous secretion of S100A8/S100A9 in WT, S100A9 KO, and CD69 KO monocytes; there was no difference in the amount of secreted S100A8/S100A9 between WT and CD69 KO monocytes, indicating that a cellular uptake mechanism by CD69 is no major pathway to influence extracellular S100A8/S100A9 concentrations. In addition, as expected, no secretion by S100A9 KO monocytes was observed (Figure 4b). Interestingly, CD69 KO monocytes presented a phenotype comparable to S100A9 KO monocytes, showing a significant reduction in expression of SOCS3, which could not be up‐regulated by further stimulation with either S100‐tetramers or S100‐dimers (Figure 4c, upper panel). Accordingly, high levels of P‐STAT3 could be detected in CD69 KO monocytes, which were not downregulated by additional S100‐tetramers or S100‐dimers (Figure 4c, lower panel). Moreover, changes in morphology with lower circularity and larger perimeters (Figure 4d), lower adhesion (Figure 4e), higher transmigration rates (Figure 4f), and migration speeds (Figure 4g) could be detected for CD69 KO cells, comparable to S100A9 KO cells, and no recovery was seen upon S100‐dimer or S100‐tetramer stimulation.

The above data suggested that CD69 could function as a specific surface receptor for S100A8/S100A9 tetramers on monocytes. To directly assess this possibility, we performed direct binding experiments with purified components by employing the quartz crystal microbalance with dissipation (QCM‐D) technique. QCM‐D measures shift in the resonance frequency of a quartz crystal that under certain conditions is proportional to the adsorbed mass; and thus, can be used to measure protein–protein interactions that result in the change of mass adsorbed to a sensor chip attached to the quartz crystal.^[^ ^27^ ^]^ To mimic a condition where CD69 is anchored in a biological membrane, we first established a solid‐supported bilayer (SLB) consisting of phosphatidylcholine (PC) and DGS‐Ni‐NTA on the surface of the sensor chip. This allowed the association of the His‐tagged extracellular domain of CD69, and thereby, the anchoring of this domain in a lipid bilayer. Next, soluble S100‐dimers or S100‐tetramers were perfused over the chip to assess potential binding by a shift in quartz crystal resonance frequency. Figure 4h‐(2) (left and right panel) shows that recombinant His‐tagged CD69 bound strongly to the Ni‐containing SLB as evidenced by the decrease in resonance frequency. Addition of S100‐tetramers led to a further decrease in frequency indicative of an additional mass adsorption and thus direct binding (Figure 4h‐(3) [left panel]). Interestingly, no such binding to the CD69 receptor was seen for S100A8/S100A9‐N70A‐dimers (Figure 4h‐(3) [right panel]). At the end of each measurement, imidazole was added to reach total desorption of the receptor from the lipid bilayer. Thus, S100‐tetramers but not S100‐dimers could interact directly with the extracellular domain of CD69. To ensure that no S100 protein bonded directly to the lipid bilayer, we performed the same experiment with and without CD69 receptor (Figure S7a,b, Supporting Information).

For correct CD69 insertion into the membrane, we performed the same experiment with a well‐known binding partner for CD69 which is galectin‐1 (Figure S7c, Supporting Information). Finally, via a titration ELISA, we determined the dissociation constant of the receptor CD69 and S100A8/S100A9‐tetramer which was in the low nanomolar range (Figure 4i).^[^ ^26^ ^]^

### 2.7 S100A8/S100A9‐Tetramers Restrict the Immune Response in a Cutaneous Granuloma Model and a Model of Irritative Contact Dermatitis In Vivo

**Figure 5:**
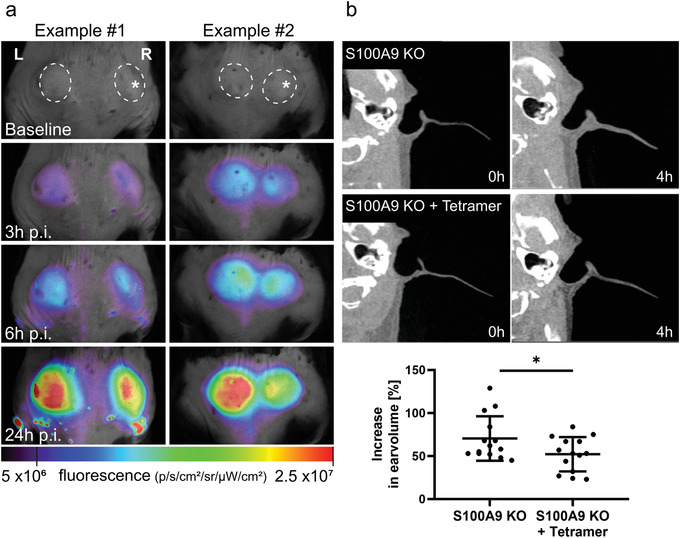
S100A8/S100A9‐tetramers dampen the immune response in inflammatory disease models. a) Time series of in vivo fluorescence reflectance imaging (FRI) in a cutaneous granuloma model. Images of two representative mice show recruitment of i.v. injected DID‐labeled monocytes to the biogel plugs (dashed circles) over time. At 24 h p.i., the LPS+/S100‐tetramer+ plugs (* label) in the right flank show a decreased cellular signal as compared to the LPS+ plug without S100‐tetramer in the left flank. Three independent experiments were performed using three mice per experiment each and the figure images of two representative mice are shown. Injection of S100‐tetramers significantly reduced immigration of monocytes (ratio tetramer‐LPS/LPS: 0.83 +/‐ 0.11, p<0.004). One‐way ANOVA with Bonferroni post‐test was used. b) Computed tomography (CT) scans of healthy (0 h) and inflamed (4 h) ears in irritant contact dermatitis (ICD). Images of two representative mice before and 4 h after ICD induction with and without tetramer application (100 µg, iv) prior induction of ICD are shown. The control mouse (upper CT pictures) shows increase of ear swelling over time which is diminished after tetramer application (lower CT pictures). The lower graph shows that the percentage of ear volume increases after 4 h of ICD induction. At 4 h p.i., S100A9 KO mice plus tetramer application show statistically significant less increase in ear volumes compared to mice without tetramer. Both ears per mouse were analyzed in three independent experiments using two, two, and three mice per experiment each and for statistical analysis, *t*‐test was used, * *P* < 0.05.

To confirm the biological relevance of our data obtained in vitro, we used a cutaneous granuloma model, in which two biogel “plugs” were injected into the right (addition of lipopolysaccharide (LPS) plus S100A8/S100A9‐tetramer) and the left flank (addition of LPS only) of the mice. The addition of LPS to both plugs served as a defined inflammatory trigger. After i.v. application of 5 × 10^6^ DID stained ER‐HoxB8‐derived monocytes, mice were imaged at times indicated in **Figure** **5**. In vivo fluorescence reflectance imaging (FRI) showed a significant decrease of cellular infiltration into tetramer/LPS plugs compared to LPS plugs 24 h after injection of the cells (ratio tetramer‐LPS/LPS: 0.83 +/− 0.11, *p* < 0.004; three independent experiments were performed using three mice per experiment each, Figure 5a). In addition, we performed a mouse model of irritative contact dermatitis (ICD). ICD was induced in S100A9 ko mice with and without application of S100A8/S100A9 tetramers (100 µg iv). Ear swelling was significantly lower in S100A9 KO mice recieving S100‐tetramers compared to controls as determined by CT scan analysis (Figure 5b).

## 3 Discussion

The results reported in this study provide evidence that S100‐dimers and S100‐tetramers exhibit distinct active and even antagonistic functions in regulating the cellular dynamics of monocytes. Our data clearly demonstrate that extracellular S100A8/S100A9 tetramer formation represents a cell autonomous mechanism keeping monocytes in a quiescent state. This finding was surprising because it has been generally accepted that the alarmin S100A8/S100A9 triggers or amplifies inflammatory responses via binding to TLR4.^[^ ^6^, ^7^, ^14^, ^28^ ^]^ Moreover, S100A8/S100A9 complexes have been described to bind to several other receptors, for example, TLR4, RAGE, CD33, CD68, CD69, CD85j, EMMPRIN (CD147), or MCAM, but none of these published interactions could explain a specific effect of the S100A8/S100A9‐tetramers.^[^ ^25^, ^29^, ^30^, ^31^, ^32^, ^33^, ^34^, ^35^ ^]^ In the presence of high extracellular calcium‐ion concentrations alone or in combination with zinc‐, nickel‐, or manganese‐ions, the S100A8/S100A9‐dimers form S100A8/S100A9‐tetramers, which mask the TLR4 binding site in the tetramer interface and block binding to TLR4.^[^ ^8^, ^15^, ^16^ ^]^ The tetramers diffuse into the systemic circulation, where they can be found in relatively high concentrations.^[^ ^5^, ^36^ ^]^ Our study now demonstrates a novel relevant biological function for these highly abundant S100‐tetramers in the absence of infectious conditions. The specific interaction of these S100A8/S100A9‐tetramers but not dimers with CD69 controls the constitutive migratory capacities of monocytes. Tetramers induced by different bivalent cations as calcium or calcium/manganese or calcium/nickel may differ in their structure and consequently, also in their function regarding binding to CD69. However, known S100A8/S100A9 tetrameric 3D‐structures in the presence of different cations are almost identical; so, we expect no functional consequences on the S100‐tetramer–CD69 interaction by binding of different cations.^[^ ^16^, ^37^, ^38^ ^]^ The analysis of wildtype S100A8/S100A8 heterodimers is not possible because their life time is too limited after release by activated phagocytes in vivo or under cell culture conditions in vitro due to the rapid calcium‐induced tetramer formation. We therefore use two independent controls for dimer‐induced effects: S100A8 homodimers^[^ ^6^, ^7^ ^]^ and S100A8/S100A9N70A heterodimers. The latter does not form tetramers due to a mutation in the calcium binding site which however does not induce significant structural changes or block inflammatory activities.^[^ ^8^, ^39^ ^]^ We confirmed the biological relevance of our in vitro findings in vivo using a cutaneous granuloma model and a model of irritative contact dermatitis. The granuloma model allows a direct quantitative comparison of the effect of S100A8/S100A9 tetramers on leukocyte recruitment triggered by a defined inflammatory stimulus within the same mouse. In the ICD model, the degree in ear swelling represents the response of mainly phagocytes to a non‐specific irritative trigger in a complex inflammatory scenario. In both inflammatory in vivo settings, S100A8/S100A9‐tetramers significantly reduce the response of immune cells even to strong inflammatory stimuli such as LPS or croton oil.

Binding of S100A8/S100A9‐tetramers to CD69 blocks STAT3 signaling via activation of SOCS3, which finally results in the control of small GTPase activities leading to a resting state in monocytes. GTPases may activate STAT3^[^ ^22^ ^]^ or STAT3 may activate GTPase activity.^[^ ^23^ ^]^ Our data of STAT3 KO cells and STAT3/S100A9 double KO cells and use of the STAT3 inhibitor Cryptotanshinone point to the latter mechanism. Signaling via other members of the STAT family is not relevant in S100A8/S100A9‐tetramer mediated effects. SOCS3 is a known inhibitor of STAT3 phosphorylation and regulator of activation in monocytes^[^ ^24^ ^]^ and STAT3 has been described to promote migration in different cell types through activation of different Rho GTPases.^[^ ^22^, ^23^, ^40^, ^41^ ^]^ It is well known that different GTPases exhibit specific effects in monocytes and macrophages. Rac and Cdc42 promote actin polymerization and generation of lamellipodial membrane protrusions at the front end of migrating cells, whereas RhoA, known to induce the formation of actin stress fibers, mediates the retraction of membrane protrusions and the trailing end of the cell.^[^ ^21^ ^]^ Accordingly, our pharmacological inhibition experiments also revealed specific effects for the individual GTPases. Uncontrolled activation of the Rho GTPase Cdc42 is the key pathway for increased filopodia formation and enhanced transmigration of S100A9 KO monocytes, whereas activation of RhoA and Rac in S100A9 KO monocytes is responsible for the enhanced basal velocity in accordance to the main functions described for these two GTPases.^[^ ^42^, ^43^ ^]^

Although CD69 was initially described as an early activation marker of lymphocytes, the receptor is also expressed on innate immune cells and also exhibits anti‐inflammatory effects.^[^ ^44^, ^45^ ^]^ For example, analysis of CD69 KO mice demonstrated a regulatory role of CD69 in attenuating inflammation and the immune response in arthritis, in pathogen clearance or tumor immunity.^[^ ^46^ ^]^ These effects have been largely attributed to lymphocytes but our data point to the fact that regulatory mechanisms induced by S100‐tetramers in monocytes could be a so far neglected pathway of CD69‐mediated regulatory effects on inflammation. In addition, S100‐tetramer binding to CD69 could affect signaling lipid sphingosine 1‐phosphate (S1P) pathway, which also may participate in the control of an appropriate immune response.^[^ ^47^, ^48^, ^49^ ^]^

S100A8 and S100A9 form intracellular dimers which are released at sites of inflammation.^[^ ^39^, ^50^ ^]^ High extracellular calcium concentrations; however, induce a shift from pro‐inflammatory S100‐dimers binding to TLR4 to regulatory S100‐tetramers with a high binding affinity to CD69 which are the predominant form in the systemic circulation.^[^ ^51^ ^]^ In fact, in a previous study, we already showed in our model of granulomatous inflammation induced by parallel injection of two subcutaneous biogel pellets (one containing LPS as inflammatory stimulus, the other PBS as control), a significantly higher abundance of S100‐dimers in the inflammatory LPS‐pellet compared to the control pellet within the same mouse.^[^ ^8^ ^]^ Thus, the same molecules drive inflammation locally but prevent spreading of inflammatory activity throughout the body. Several mechanisms have been described which may contribute to the stabilization of the dimeric form of S100A8/S100A9. Low pH values, which is a common finding at sites of inflammation, shift the equilibrium to S100‐dimers; and thus, promote inflammatory processes.^[^ ^8^ ^]^ Accordingly, analysis of local inflammatory exudates with a high abundance of S100A8/A9 shows indeed conditions which favor a shift from tetramers to dimers, that is, low calcium and lower pH.^[^ ^52^, ^53^ ^]^ In addition, reactive oxygen molecules released by phagocytes at sites of inflammation may lead to oxidation of S100A9 which has been described to inhibit tetramer formation as well.^[^ ^54^ ^]^ As tetramer formation is also induced by zinc ions, our findings may also present a novel mechanism explaining why zinc deficiency promotes inflammatory processes and tissue damage.^[^ ^55^, ^56^ ^]^ The high systemic concentrations of S100A8/S100A9 tetramers are thus not simply an inactive byproduct as supposed so far but an important buffer protecting from harmful spreading of inflammatory processes which may be of high relevance because S100A8 and S100A9 are the most abundant alarmins in for example, infections, autoimmune, or cardiovascular diseases.^[^ ^5^, ^57^ ^]^ However, the relevance of this mechanism in specific inflammatory diseases is currently difficult to assess because reliable methods to quantify the different complex forms are still lacking. Environmental factors regulating dimer/tetramer ratios at local sites of inflammation may differ significantly under specific inflammatory conditions, tissues, exudates, granulomas, or even abscesses. Quantitative data about local calcium and zinc concentrations, pH, or even ROS production are astonishingly rare for most inflammatory diseases. Differences in these factors may explain some organ specific impact on the beneficial or destructive consequences of inflammation. Future approaches need to address the question of under which inflammatory conditions environmental factors in tissues such as ions, pH or ROS decrease to retard the formation of S100 tetramers, and thereby may promote unwanted inflammation.

**Figure 6:**
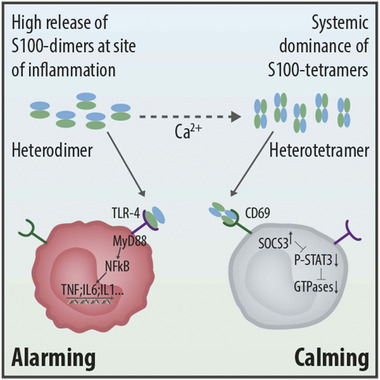
Alarming and calming: Functions of S100A8/S100A9 dimers and tetramers on monocytes. High release of S100A8/S100A9‐dimers at local sites of inflammation leads to the well‐known activation of monocytes via binding to TLR4 (left cell: alarming). However, calcium‐induced S100A8/S100A9‐tetramer formation, which occurs rapidly in the extracellular space, blocks the TLR4‐binding site restricting their inflammatory activities. S100A8/S100A9‐tetramers keep monocytes in a steady state condition via a CD69 receptor mediated mechanism (right cell: calming). Thus, due to their calcium‐induced quaternary structure, S100A8 and S100A9 show local and systemic antagonistic functions due to interactions with different receptors.

Changes in a quaternary structure have often been described to regulate the activity of proteins, in particular, enzymatic activities, protein–protein interactions, or the assembly of multi‐protein complexes. However, in this case, we have the unique feature of a quaternary structure not simply switching the activity of protein complexes “on” or “off” but rather inducing a shift from binding and activating a pro‐inflammatory receptor (TLR4) to an opposite regulatory function mediated specifically by a different receptor (CD69) (**Figure** **6**), a situation which to our knowledge has not been described so far.

## 4 Experimental Section

### Animals

S100A9‐/‐ mice (9–15 weeks old, C57BL/6 background) were bred and housed in individually ventilated cages. Of note, S100A9‐/‐ mice are double deficient for S100A9 and S100A8 at the protein level.^[^ ^8^, ^19^ ^]^ The animal housing conditions and animal experiments were approved by the local authorities (Landesamt für Natur, Umwelt und Verbraucherschutz Nordrhein‐Westfalen register no. 81‐02.04.2018.A103). For generation of ER‐Hoxb8 cells male C57BL/6 wild‐type, S100A9 knockout, CD69 knockout, TLR4 knockout, and RAGE knockout as well as EGFP‐Lifeact wild‐type and EGFP‐Lifeact S100A9 knockout mice were maintained in specific pathogen free conditions at the animal facility of the Institute of Immunology Muenster.

### Isolation of Murine Bone Marrow Cells

Bone marrow cells (BMC) were isolated from femur and tibia by flushing with PBS containing 1% FCS (PBS/FCS). After lysis of erythrocytes and washing in PBS/FCS, cells were differentiated for up to 7 days in RPMI1640 medium supplemented with 10% FCS, 1% Pen/Strep, 1% L‐Glutamine, and 20% supernatant of M‐CSF expressing L‐929 cells (Lonza).

### Cells

Generation of ER‐Hoxb8 cell lines were made as previously described (Wang et al., 2006). Cells were cultured in RPMI1640 Medium (Merck Millipore) supplemented with 10% FCS (Pan Biotech), 1% Pen/Strep (Biochrom), 1% L‐Glutamine (Biochrom), 2 µg recombinant granulocyte‐macrophage colony‐stimulating factor (GM‐CSF) (Immunotools), and 1 µm *β*‐Estradiol (Sigma–Aldrich). For differentiation to monocytes, cells were washed twice in PBS containing 1% FCS to remove *β*‐Estradiol. Cells were differentiated for up to 7 days in RPMI1640 medium supplemented with 10% FCS, 1% Pen/Strep, 1% L‐Glutamine, and 20% supernatant of M‐CSF expressing L‐929 cells (Lonza). Of note, the final calcium concentration in the differentiation medium was ≈0.89 mmol L^−1^.

### Preparation of S100 Proteins

Murine S100A8, S100A9, and S100A9‐N70A proteins were prepared according to the protocol for the human S100 proteins. Briefly, murine S100A8, S100A9, and S100A9‐N70A proteins were cloned in pET1120 vector, expressed in E.coli BL21 bacteria and purified from inclusion bodies. S100 containing inclusion bodies were purified and solubilized in 8 m urea in the presence of 10 mm DTT. Renaturation was performed by stepwise increase of the pH from 2 to 4.7 and 7.4 followed by two chromatography runs (Äcta‐FPLC, anion exchange, and size exclusion chromatography). Pure fractions of the S100 proteins were collected, dialized in HBS, and frozen in aliquots before use. Heterodimers were prepared by mixing equal amounts of S100A8 and S100A9 or S100A8 and S100A9‐N70A in the presence of 1 mm DTT followed by a denaturation step at pH 2. Renaturation was again achieved by stepwise increase of the pH to 7.4. Due to that, heterodimers were much more stable compared to homodimers; the formation of heterodimers is preferred. Possible residual contaminating homodimers in the preparations were separated by anion exchange chromatography runs (Äcta FPLC). Fractions containing only heterodimers were collected, aliquoted, and frozen before use. Tetramers were induced by adding calcium (1 mm). The identity of all preparations was tested by western blot and functional activity measurements and only those batches were used which were entirely free of any LPS contaminations.^[^ ^6^, ^7^, ^8^, ^55^ ^]^

### Cell Culture

Cells were stimulated or treated with different inhibitors as indicated in the figures. As stimuli, the complement C5a (20 nm; R&D) as well as purified S100A8/S100A8‐homodimer (250 ng mL^−1^), S100A8/S100A9‐heterodimer (250 ng mL^−1^), and the mutated S100A8/S100A9‐N70A‐heterodimer (250 ng mL^−1^) were used. For the inhibition of the Rho GTPases, RhoA, Rac, and Cdc42, the cells were treated with the specific GEF inhibitors Rhosin hydrochloride (30 µm), W56 (250 µm), and ZCL278 (50 µm) for 30 min, respectively (Tocris). Cryptotanshinone was used to inhibit the phosphorylation of STAT3 at a concentration of 5 µm for 30 min (Sigma–Aldrich).

### Adhesion Assay

2 × 10^5^ cells were seeded on 50 µg mL^−1^ fibronectin (Sigma–Aldrich) coated 96 well plates and allowed to adhere for 30 min at 37 °C under 5% CO_2_. The optical density of the adhering cells was measured at 560 nm wavelength after having fixed the adherent cells with 2% glutaraldehyde (Roth) for 10 min, stained with 0.5 % crystal violet, and lysed with 10 % acetic acid (Roth). Quantification was obtained via optical density of crystal violet stained adherent cells.

### Transmigration Assay

A boyden chamber assay was used to investigate the transmigration properties of the cells. The two chambers were separated by a porous membrane (5 µm pore size, ThermoFisher Scientific). The lower chamber was filled either with medium or medium containing the chemoattractant (complement C5a, 20 nm) and in the upper chamber, 1 × 10^6^ cells were seeded. After six hours at 37 °C under 5 % CO_2_ the transmigrated cells in the lower chamber were harvested and counted with a cell counter (Schärfe System).

### Western Blot

Cells were lysed with a Mammalian Protein Extraction Reagent lysis buffer (ThermoFisher Scientific) containing a protease inhibitor cocktail (Roche) and phosphatase inhibitor cocktail (Santa Cruz). Proteins were separated according to the protein size by a 10% or 15% SDS‐PAGE and transferred onto a nitrocellulose membrane (ThermoFisher Scientific). Membranes were blocked for 1 h at room temperature in TBST containing 10% milk powder (Roth) followed by overnight incubation with primary antibodies diluted in TBST containing 5% bovine serum albumin (Roth) at 4 °C. For detection, horseradish peroxidase‐conjugated secondary antibody (Cell Signaling) diluted in TBST containing 5% milk powder was used in combination with enhanced chemiluminescence (ECL) solution to detect the bands via a Chemidoc XRS^+^ (BioRad). For statistical analysis, the intensity of the bands was measured via ImageJ and the quantification of STAT3 activation corresponded to the ratio of P‐STAT3 on total STAT3 levels after normalization to their GAPDH bands and the quantification of SOCS3 corresponded to the ratio of SOCS3 levels after normalization to GAPDH levels.

### Immunofluorescence

EGFP‐Lifeact ER‐Hoxb8 WT and EGFP‐Lifeact ER‐Hoxb8 S100A9 KO monocytes were differentiated on coverslips for 3 days and afterward treated as indicated in the figures. Cells were fixed with 4% of PFA for 30 min, permeabilized with 0.1% of TritonX for 10 min, and incubated overnight with the primary antibody P‐STAT3. Cells were washed and incubated with a secondary antibody conjugated to Cy3 for 30 min and subsequently, the coverslips were mounted on a slide. For statistical analysis, the intensity of the P‐STAT3 signal was measured via ImageJ in the nucleus and normalized to the signal in the cytoplasm.

### Immunoprecipitation

EGFP‐Lifeact ER‐Hoxb8 WT and EGFP‐Lifeact ER‐Hoxb8 S100A9 KO monocytes were treated and analyzed for the GTP‐bound RhoA, Rac, and Cdc42 according to the manufacturer's protocol (Cytoskeleton, Inc.). Immunoprecipitated GTPases (GTP‐bound) and total amounts were analyzed by western blot. The intensity of the bands was quantified using ImageJ and the GTPase activities = relative intensities shown in the lower bar graphs corresponded to the ratio of GTP bound GTPase (IP:GTPase) to total GTPase and for total GTPases, a GAPDH blot was performed as a loading control.

### Flow Cytometry

Surface expression of antigens was performed after washing of cells with PBS/FCS. Cells were incubated for 30 min in 4 °C with fluorescence‐labeled antibodies. After washing, cells were measured or fixed in 2% PFA at 4 °C for 20 min prior to intracellular staining. PFA fixed cells were permeabilized (BD Bioscience) at 4 °C for 20 min. Intracellular staining was performed with the desired antibodies at 4 °C for 30 min. After washing, cells were measured on a Navios flow cytometer (Beckman Coulter). Flow cytometer and data were analyzed using FlowJo software (Tree Star).

### ELISA

Concentrations of S100A8/S100A9 in culture medium were quantified by a sandwich ELISA system established in our laboratory as described previously.^[^ ^58^ ^]^

### Quartz Crystal Microbalance Dissipation (QCM‐D)

1,2‐dioleoyl‐sn‐glycero‐3‐phosphocholine (DOPC) and 1,2‐dioleoyl‐sn‐glycero‐3‐[(N‐(5‐amino‐1‐ carboxypentyl)iminodiacetic acid)succinyl] Ni salt (DGS‐Ni‐NTA) were purchased from Avanti Polar Lipids Inc. (Alabaster, AL). All other chemical/solvents were purchased from Merck, Carl Roth, or AppliChem (Darmstadt, Germany). For the experiments, recombinant mouse CD69 (250 nm; R&D) as well as purified S100A8/S100A9‐heterodimer (1 µg mL^−1^), and the mutated S100A8/S100A9‐N70A‐heterodimer (1 µg mL^−1^) were used.

*Liposome Preparation*: To generate small unilamellar vesicles (SUV), the respective lipids were dissolved in chloroform/methanol (1:1 v/v) and mixed to obtain the composition DOPC/DGS‐Ni‐NTA of 96:4. The organic solvents were then removed under a stream of nitrogen above the lipid gel–fluid phase transition temperature. Residual traces of the organic solvent were evaporated in high vacuum for 1 h at the same temperature and lipid films were stored at 4 °C until use. Dry lipid films were resuspended in citrate buffer [10 mm trisodium citrate (Merck, Darmstadt, Germany) and 150 mm NaCl, pH = 4.6 (at RT)] at 60 °C and kept for 30 min with steps of vortex mixing every 5 min resulting in the formation of multilamellar vesicles (MLVs). To obtain SUVs, the MLVs were extruded 21 times using a polycarbonate membrane with a pore size of 50 nm (Avanti Polar Lipids Inc., Alabaster, AL).

*Supported Lipid Bilayer (SLBs)*: QCM‐D Quartz sensors (QSX 303, 50 nm SiO_2_, 4.95 MHz) were cleaned in 2% (w/v) SDS and hydrophilized by a 10‐min O_2_‐plasma treatment (Harrick Plasma, Ithaca, NY). Subsequently, surfaces were rinsed with ultra‐pure water and dried in a stream of nitrogen. To prepare the supported bilayer membrane on the quartz sensor by vesicle rupture, SUVs (0.5 mg mL^−1^, 50 nm) were applied in the QCM‐D loop‐flow mode. Following membrane formation, the citrate buffer was exchanged to a HBS buffer (10 mm HEPES and 150 mm NaCl, pH of 7.4 [at RT], containing CaCl_2_, EGTA or imidazole, as indicated) prior to the protein solution.

*QCM‐D Measurements*: Quartz crystal microbalance measurements with dissipation (QCM‐D) were performed on a Q‐Sense E4 QCM‐D Analyzer (Q‐Sense, Gothenburg, Sweden) equipped with four temperature‐controlled flow cells in a parallel configuration. Flow cells were connected to a peristaltic pump (Ismatec IPC, Glattbrugg, Switzerland) employing a flow rate of 80.4 µL min^−1^. Binding analysis was performed at 20 °C in HBS Buffer supplemented with 250 µm CaCl_2_. Frequency and dissipation shifts (7th overtone) of the quartz sensor (QSX 303, 50 nm SiO2, 4.95 MHz) were monitored and considered for data evaluation. Calculations were carried out using OriginPro v. 9.1 (OriginLab Corp., Northampton, MA).

### Titration ELISA

A 10 nm S100A8/S100A9‐tetramer concentration (HBS plus 250 µm CaCl_2_) was coated to the bottom of a 96‐well plate. After three washing steps, unspecific binding sites were blocked by 0.25% BSA/HBS plus 250 µm CaCl_2_. Afterward, His‐tagged‐CD69 (R&D) was added at increasing concentrations as indicated in the figure. After 1 h, unbound CD69 was removed by three washing steps and an anti‐His‐antibody (10 µg mL^−1^, Thermo Fisher) was added in excess to quantify bound CD69. The plate was again washed for three times followed by the addition of a secondary horseradish peroxidase (HRP) coupled antibody (Cell Signaling). Afterward, a peroxidase substrate solution (TMB; GE Healthcare) was dispensed to the plate and after 15 min of incubation, the stop solution H_2_SO_4_ (2N) was added to each well. The absorbance at 540 nm was measured using an ELISA reader (Anthos). A non‐linear regression analysis was performed to calculate the binding constant of CD69 to S100‐tetramer assuming a 1:1 binding model of one S100A8/S100A9‐tetramer binding one CD69‐dimer.^[^ ^26^ ^]^

### 2D Migration Assay

EGFP‐Lifeact ER‐Hoxb8 monocytes were seeded into a narrow channel of a fibronectin coated µ‐slide chemotaxis chamber (Ibidi) which is connected with two reservoirs. After 30 min, the chemotaxis chamber was filled with RPMI 1640 (bicarbonate) medium plus 10% FCS and 1% Pen/Strep. The reservoirs were filled with medium containing 0.003% Patent Blue V (Sigma–Aldrich) (blue dye) either with or without complement C5a (20 nm). The observation area was imaged with an inverted Axio Observer.Z1 epifluorescence microscope (Zeiss) with 10×/0.25 objective. Images were acquired every minute for up to 6 h and analyzed with ImageJ (National Institutes of Health) using a manual tracking plugin and the chemotaxis and migration tool from Ibidi.

### Spinning Disk Microscopy

EGFP‐Lifeact ER‐Hoxb8 cells were seeded into a narrow channel of a fibronectin coated µ‐slide chamber (Ibidi) for 30 min. ER‐Hoxb8 WT, S100A9 knockout, TLR4 knockout, RAGE knockout and CD69 knockout monocytes were seeded on coverslips, fixed (4% PFA), and labeled with Alexa Fluor 488‐phalloidin (ThermoFisher Scientific). Images were taken with an inverted Nikon Eclipse Ti‐e equipped with a Spinning Disk module (Yokogawa, W1). The circularity and perimeter of the cells were determined by ImageJ.

For the calcium measurements, EGFP‐Lifeact ER‐Hoxb8 cells were seeded into a narrow channel of a fibronectin coated µ‐slide chamber (Ibidi) to let adhere for 30 min. The calcium dye Cal590AM (Biomol) was dissolved in Pluronic F‐127/DMSO (Invitrogen). 4 µL of this solution was mixed with 4 mL RPMI 1640 (bicarbonate) and supplemented with 0.5 mm Probenecid (Santa Cruz). The reservoirs connected to the channel were filled with the dye mix and after 20 min at 37 °C under 5% CO_2_, cells were washed with medium and imaged. Time‐lapse imaging was performed using an UltraVIEW Vox 3D live cell imaging system (Perkin Elmer) coupled to a Nikon Eclipse Ti inverted microscope. The system incorporated a Yokogawa (Japan) CSU‐X1 spinning disk scanner, a Hamamatsu (Japan) C9100‐50 EM‐CCD camera (1000 × 1000 pixels), and Volocity software. Cells were imaged every 10 s for up to 6 min and after the first minute of imaging complement C5a (20 nm) was added. The temperature was maintained at 37 °C using an Okolab all‐in‐one stage incubator (Okolab, Ottavianio) and images were taken via a Nikon 60x/1.49 oil immersion objective. The Ca^2+^ intensity signal was measured over time via ImageJ.

### Traction Force Microscopy (TFM)

Glass bottom dishes were pretreated with 200 µL APTMS washed and incubated for 30 min with 0.5% glutaraldehyde. A pre‐mix of 500 µL acrylamide plus 4 µL acrylic acid and 250 µL bisacrylamide was prepared. To 75 µL of the pre‐mix, 415 µL 65% PBS, 10 µL polystyrene beads (100 nm; Micromod), 1.5 µL TEMED (Sigma–Aldrich), and 5 µL APS (Roth) were added. 5 µL of this gel mix was pipetted onto the glass bottom dish, covered with a coverslip. After solidification, the gel was covered with 65% PBS and the coverslip was removed. 100 µL of 50 µg EDC (Roth) dissolved in 1 mL NHS/NaCl stock solution was pipetted on top of the gel for 15 min. After washing the gels with PBS, they were incubated with 50 µg mL^−1^ fibronectin (Sigma–Aldrich) for 1 h at 37 °C under 5% CO_2_. Day 3 differentiated cells treated as indicated in the figures were seeded on the gels and after 30 min, the gels were flushed with RPMI 1640 (bicarbonate) and imaged. TFM was performed at 37 °C with an inverted Nikon Eclipse Ti‐e microscope equipped with a spinning disk unit (Yokogawa, W1), 60×/1.2 objective and 488 nm and 561 nm lasers. A *z*‐stack of 5 µm (plane distance 0.33 µm) was taken every 5 s for 15 min, employing the perfect focus system (Nikon). After 15 min, 10% SDS was added and a null force image was taken. The traction force analysis was performed with a custom written MATLAB program.^[^ ^20^ ^]^

### Cutaneous Granuloma Model (CG)

The cutaneous granuloma model was performed as previously described.^[^ ^50^ ^]^ Briefly, mice where shaved and 200 µL of Biogel (P‐100, Bio Rad, Hercules, USA) was injected as a “plug” subcutaneously at both flanks of the mice. Both biogels contained 1 µg/200 µL lipopolysaccharide (LPS from Salmonella enterica serotype enteritidis, Sigma–Aldrich, Munich, Germany) as inflammatory trigger. 100 µg/200 µL S100A8/S100A9 tetramers were added only to the right plug (seen from the dorsal surface). FRI measurements were performed at day one after injection of DID labeled ER‐HoxB8 cells at baseline: 3, 6, and 24 h.

### Eliciting of Irritant Contact Dermatitis (ICD)

ICD was induced by application of 3% croton oil in olive oil‐acetone (1:4) to the dorsal surface of both ears of mice (7 per group). S100A9 deficient mice were chosen for these experiments which allowed to control potentially confounding effects of endogenously released S100A8/S100A9. S100A9ko mice were divided into two groups and prior to induction, one group received additional 100 µg of S100A8/S100A9 tetramers intravenously. Prior to *t* = 0 h and 4 h after ICD induction, CT scans were performed to evaluate the ear volumes.

### Labeling

ER‐HoxB8 cells differentiated for 3 days toward monocytes were harvested and 1 × 10^6^ cells per mL were labeled with 5 µmol/l 1,1‐Dioctadecyl‐3,3,3,3‐tetramethylindodicarbocyanine (DID)/EtOH (ThermoFisher Scientific, Waltham, USA) for 5 min and washed three times. Labeling efficiency was checked by flow cytometry, and viability controlled by trypan blue or 7AAD staining. For in vivo FRI experiments, 5 × 10^6^ labeled cells/200 µL PBS were injected (i.v.) into the lateral tail vein.^[^ ^50^ ^]^

### FRI Measurements

In vivo imaging of labeled ER‐HoxB8 cells by FRI in the CG model was carried out using the IVIS Spectrum system (epi‐illumination) by PerkinElmer, Waltham, USA (605 nm excitation/680 nm emission for DID). Identical illumination settings (exposure time: automatic, lamp voltage = 21 V, f‐stop = 2, field of view = B [6.6 cm], and binning = 2) were used for all experiments. Mice were kept under anesthesia (DRÄGER Isofluran Vapor, Lübeck, Germany) and kept warm during the imaging process. Fluorescence images were analyzed by using in‐house software MEDgical. Fluorescence emission was measured by fluorescence emission radiance per incident excitation irradiance (radiant efficiency) in p/s/cm2/sr/µW/cm2. Grayscale photoimages and fluorescence color images were overlaid.

### Computed Tomography (CT) Scanning

Animals were anesthetized with isoflurane and placed on a heat controlled multimodal scanning bed and transferred to the computed tomography scanner (Inveon, Siemens Medical Solutions, USA) and a medium resolution (25 µm) computed tomography acquisition was performed for each mouse. Computed tomography was reconstructed into a volume data set with a voxel size of 0.09×0.09×0.09 mm^3^. An inhouse‐developed software MEDgical was used to segment and quantify the volumes of the outer ear.

### Quantification and Statistical Analysis

Statistical analysis was performed with Graph Pad Prism Software using the appropriate tests according to normal or non‐normal data distribution: unpaired *t*‐test (Figures 1e, 4a, and 5b), Mann‐Whitney U test (Figures 1d and 2k), and Wilcoxon Signed Rank Test. Three independent experiments were performed using three mice per experiment each (Figure 5a) and One‐way ANOVA with Bonferroni post‐test (for all other figures). *P* values were derived from one‐way ANOVA with Bonferroni post‐test analysis unless otherwise noted. Error bars denote the SD. Statistical significance was defined as *p* < 0.05. All quantitative data shown using representative data were repeated in at least three independent replications.

## Conflict of Interest

The authors declare no conflict of interest.

## Author Contributions

Designed and performed experiments, analyzed results, and wrote the manuscript: A.R. Performed experiments and provided inputs on the manuscript: H.S., M.B., A.L.L.M., D.G., M.v.W., L.F.‐R., P.J.H., and S.H. Provided access to the Hoxb8 vector, analyzed data, and provided inputs on the manuscript: H.H. Provided EGFP‐Lifeact WT and EGFP‐Lifeact S100A9 knockout mice and inputs on the manuscript: M.S. Provided access to the CD69 knockout mice and input on the manuscript: F.S.‐M. Performed revision experiments: K.S., J.R., M.R., and L.K. Designed and supervised experiments, discussed data, and provided input on the manuscript: V.G. and T.B. Conceived and supervised the study, designed experiments, discussed data, and wrote the manuscript: T.V. and J.R.

## Supporting information

Supporting Information

## Data Availability

The data that support the findings of this study are available from the corresponding author upon reasonable request.

## References

[1] F. Ginhoux , S. Jung , Nat. Rev. Immunol. 2014, 14, 392. 24854589 10.1038/nri3671

[2] M. Guilliams , A. Mildner , S. Yona , Immunity 2018, 49, 595. 30332628 10.1016/j.immuni.2018.10.005

[3] K. Odink , N. Cerletti , J. Brüggen , R. G. Clerc , L. Tarcsay , G. Zwadlo , G. Gerhards , R. Schlegel , C. Sorg , Nature 1987, 330, 80. 3313057 10.1038/330080a0

[4] A. Rammes , J. Roth , M. Goebeler , M. Klempt , M. Hartmann , C. Sorg , J. Biol. Chem. 1997, 272, 9496. 9083090 10.1074/jbc.272.14.9496

[5] J. Austermann , C. Spiekermann , J. Roth , Nat. Rev. Rheumatol. 2018, 14, 528. 30076385 10.1038/s41584-018-0058-9

[6] T. Vogl , K. Tenbrock , S. Ludwig , N. Leukert , C. Ehrhardt , M. A. van Zoelen , W. Nacken , D. Foell , T. van der Poll , C. Sorg , J. Roth , Nat. Med. 2007, 13, 1042. 17767165 10.1038/nm1638

[7] S. K. Fassl , J. Austermann , O. Papantonopoulou , M. Riemenschneider , J. Xue , D. Bertheloot , N. Freise , C. Spiekermann , A. Witten , D. Viemann , S. Kirschnek , M. Stoll , E. Latz , J. L. Schultze , J. Roth , T. Vogl , J. Immunol. 2015, 194, 575. 25505274 10.4049/jimmunol.1401085

[8] T. Vogl , A. Stratis , V. Wixler , T. Völler , S. Thurainayagam , S. K. Jorch , S. Zenker , A. Dreiling , D. Chakraborty , M. Fröhling , P. Paruzel , C. Wehmeyer , S. Hermann , O. Papantonopoulou , C. Geyer , K. Loser , M. Schäfers , S. Ludwig , M. Stoll , T. Leanderson , J. L. Schultze , S. König , T. Pap , J. Roth , J. Clin. Invest. 2018, 128, 1852. 29611822 10.1172/JCI89867 PMC5919817

[9] J. A. Tibble , I. Bjarnason , World J. Gastroenterol. 2001, 7, 460. 11819811 10.3748/wjg.v7.i4.460 PMC4688655

[10] H. B. Schonthaler , J. Guinea‐Viniegra , S. K. Wculek , I. Ruppen , P. Ximénez‐Embún , A. Guío‐Carrión , R. Navarro , N. Hogg , K. Ashman , E. F. Wagner , Immunity 2013, 39, 1171. 24332034 10.1016/j.immuni.2013.11.011

[11] S. Wang , R. Song , Z. Wang , Z. Jing , S. Wang , J. Ma , Front. Immunol. 2018, 9, 1298. 29942307 10.3389/fimmu.2018.01298 PMC6004386

[12] T. Vogl , J. Roth , C. Sorg , F. Hillenkamp , K. Strupat , J. Am. Soc. Mass Spectrom. 1999, 10, 1124. 10536818 10.1016/s1044-0305(99)00085-9

[13] K. Strupat , H. Rogniaux , A. Van Dorsselaer , J. Roth , T. Vogl , J. Am. Soc. Mass Spectrom. 2000, 11, 780. 10976885 10.1016/S1044-0305(00)00150-1

[14] J. K. Chan , J. Roth , J. J. Oppenheim , K. J. Tracey , T. Vogl , M. Feldmann , N. Horwood , J. Nanchahal , J. Clin. Invest. 2012, 122, 2711. 22850880 10.1172/JCI62423 PMC3408740

[15] E. M. Zygiel , E. M. Nolan , Annu. Rev. Biochem. 2018, 87, 621. 29925260 10.1146/annurev-biochem-062917-012312 PMC6066180

[16] S. M. Damo , T. E. Kehl‐Fie , N. Sugitani , M. E. Holt , S. Rathi , W. J. Murphy , Y. Zhang , C. Betz , L. Hench , G. Fritz , E. P. Skaar , W. J. Chazin , Proc. Natl. Acad. Sci. U. S. A. 2013, 110, 3841. 23431180 10.1073/pnas.1220341110 PMC3593839

[17] G. G. Wang , K. R. Calvo , M. P. Pasillas , D. B. Sykes , H. Häcker , M. P. Kamps , Nat. Methods 2006, 3, 287. 16554834 10.1038/nmeth865

[18] J. A. Hobbs , R. May , K. Tanousis , E. McNeill , M. Mathies , C. Gebhardt , R. Henderson , M. J. Robinson , N. Hogg , Mol. Cell. Biol. 2003, 23, 2564. 12640137 10.1128/MCB.23.7.2564-2576.2003 PMC150714

[19] M. P. Manitz , B. Horst , S. Seeliger , A. Strey , B. V. Skryabin , M. Gunzer , W. Frings , F. Schönlau , J. Roth , C. Sorg , W. Nacken , Mol. Cell. Biol. 2003, 23, 1034. 12529407 10.1128/MCB.23.3.1034-1043.2003 PMC140712

[20] T. Betz , D. Koch , Y. B. Lu , K. Franze , J. A. Käs , Proc. Natl. Acad. Sci. U. S. A. 2011, 108, 13420. 21813757 10.1073/pnas.1106145108 PMC3158236

[21] A. J. Ridley , M. A. Schwartz , K. Burridge , R. A. Firtel , M. H. Ginsberg , G. Borisy , J. T. Parsons , A. R. Horwitz , Science 2003, 302, 1704. 14657486 10.1126/science.1092053

[22] M. Debidda , L. Wang , H. Zang , V. Poli , Y. Zheng , J. Biol. Chem. 2005, 280, 17275. 15705584 10.1074/jbc.M413187200

[23] T. S. Teng , B. Lin , E. Manser , D. C. Ng , X. Cao , J. Cell Sci. 2009, 122, 4150. 19861492 10.1242/jcs.057109

[24] R. Lang , A. L. Pauleau , E. Parganas , Y. Takahashi , J. Mages , J. N. Ihle , R. Rutschman , P. J. Murray , Nat. Immunol. 2003, 4, 546. 12754506 10.1038/ni932

[25] C. R. Lin , T. Y. Wei , H. Y. Tsai , Y. T. Wu , P. Y. Wu , S. T. Chen , FASEB J. 2015, 29, 5006. 26296369 10.1096/fj.15-273987

[26] J. A. Eble , J. Vis. Exp. 2018, 15, 57334.

[27] J. Rickert , A. Brecht , W. Göpel , Anal. Chem. 1997, 69, 1441. 21639350 10.1021/ac960875p

[28] K. Loser , T. Vogl , M. Voskort , A. Lueken , V. Kupas , W. Nacken , L. Klenner , A. Kuhn , D. Foell , L. Sorokin , T. A. Luger , J. Roth , S. Beissert , Nat. Med. 2010, 16, 713. 20473308 10.1038/nm.2150

[29] V. Arnold , J. S. Cummings , U. Y. Moreno‐Nieves , C. Didier , A. Gilbert , F. Barré‐Sinoussi , D. Scott‐Algara , Retrovirology 2013, 10, 1742. 10.1186/1742-4690-10-122 PMC3826667 24156302

[30] T. Hibino , M. Sakaguchi , S. Miyamoto , M. Yamamoto , A. Motoyama , J. Hosoi , T. Shimokata , T. Ito , R. Tsuboi , N. H. Huh , Cancer Res. 2013, 73, 172. 23135911 10.1158/0008-5472.CAN-11-3843

[31] U. Y. Moreno‐Nieves , C. Didier , Y. Lévy , F. Barré‐Sinoussi , D. Scott‐Algara , Front Immunol 2015, 6, 478. 26441983 10.3389/fimmu.2015.00478 PMC4585218

[32] A. A. Basiorka , K. L. McGraw , E. A. Eksioglu , X. Chen , J. Johnson , L. Zhang , Q. Zhang , B. A. Irvine , T. Cluzeau , D. A. Sallman , E. Padron , R. Komrokji , L. Sokol , R. C. Coll , A. A. Robertson , M. A. Cooper , J. L. Cleveland , L. A. O'Neill , S. Wei , A. F. List , Blood 2016, 128, 2960. 27737891 10.1182/blood-2016-07-730556 PMC5179338

[33] K. Okada , S. Arai , H. Itoh , S. Adachi , M. Hayashida , H. Nakase , M. Ikemoto , J. Leukocyte Biol. 2016, 100, 1093. 27312849 10.1189/jlb.2A0415-170RRR

[34] M. Sakaguchi , M. Yamamoto , M. Miyai , T. Maeda , J. Hiruma , H. Murata , R. Kinoshita , I. M. Winarsa Ruma , E. W. Putranto , Y. Inoue , S. Morizane , N. H. Huh , R. Tsuboi , T. Hibino , J. Invest. Dermatol. 2016, 136, 2240. 27388991 10.1016/j.jid.2016.06.617

[35] Y. Chen , I. W. Sumardika , N. Tomonobu , R. Kinoshita , Y. Inoue , H. Iioka , Y. Mitsui , K. Saito , I. M. W. Ruma , H. Sato , A. Yamauchi , H. Murata , K. I. Yamamoto , S. Tomida , K. Shien , H. Yamamoto , J. Soh , J. Futami , M. Kubo , E. W. Putranto , T. Murakami , M. Liu , T. Hibino , M. Nishibori , E. Kondo , S. Toyooka , M. Sakaguchi , Neoplasia 2019, 21, 627. 31100639 10.1016/j.neo.2019.04.006 PMC6520639

[36] D. Holzinger , K. Tenbrock , J. Roth , Front. Immunol. 2019, 10, 182. 30828327 10.3389/fimmu.2019.00182 PMC6384255

[37] I. P. Korndörfer , F. Brueckner , A. Skerra , J. Mol. Biol. 2007, 370, 887. 17553524 10.1016/j.jmb.2007.04.065

[38] T. G. Nakashige , E. M. Zygiel , C. L. Drennan , E. M. Nolan , J. Am. Chem. Soc. 2017, 139, 8828. 28573847 10.1021/jacs.7b01212 PMC5754018

[39] N. Leukert , T. Vogl , K. Strupat , R. Reichelt , C. Sorg , J. Roth , J. Mol. Biol. 2006, 359, 961. 16690079 10.1016/j.jmb.2006.04.009

[40] R. G. Hodge , A. J. Ridley , Small GTPases 2020, 11, 8. 29189096 10.1080/21541248.2017.1362495 PMC6959303

[41] Y. R. Pan , C. C. Chen , Y. T. Chan , H. J. Wang , F. T. Chien , Y. L. Chen , J. L. Liu , M. H. Yang , Nat. Commun. 2018, 9, 018. 10.1038/s41467-018-06134-z PMC6135800 30209389

[42] R. Horwitz , D. Webb , Curr. Biol. 2003, 13, 014. 10.1016/j.cub.2003.09.014 14521851

[43] M. Vicente‐Manzanares , F. Sánchez‐Madrid , Nat. Rev. Immunol. 2004, 4, 110. 15040584 10.1038/nri1268

[44] S. F. Ziegler , F. Ramsdell , M. R. Alderson , Stem Cells 1994, 12, 456. 7804122 10.1002/stem.5530120502

[45] B. León , G. Martínez del Hoyo , V. Parrillas , H. H. Vargas , P. Sánchez‐Mateos , N. Longo , M. López‐Bravo , C. Ardavín , Blood 2004, 103, 2668. 14630812 10.1182/blood-2003-01-0286

[46] D. Cibrian , F. Sanchez‐Madrid , Eur. J. Immunol. 2017, 47, 946. 28475283 10.1002/eji.201646837 PMC6485631

[47] L. R. Shiow , D. B. Rosen , N. Brdickova , Y. Xu , J. An , L. L. Lanier , J. G. Cyster , M. Matloubian , Nature 2006, 440, 540. 16525420 10.1038/nature04606

[48] A. A. L. Baeyens , S. R. Schwab , Annu. Rev. Immunol. 2020, 38, 759. 32340572 10.1146/annurev-immunol-081519-083952

[49] A. Baeyens , S. Bracero , V. S. Chaluvadi , A. Khodadadi‐Jamayran , M. Cammer , S. R. Schwab , Nature 2021, 592, 290. 33658712 10.1038/s41586-021-03227-6 PMC8475585

[50] S. Gran , L. Honold , O. Fehler , S. Zenker , S. Eligehausen , M. T. Kuhlmann , E. Geven , M. van den Bosch , P. van Lent , C. Spiekermann , S. Hermann , T. Vogl , M. Schäfers , J. Roth , Theranostics 2018, 8, 2407. 29721088 10.7150/thno.23632 PMC5928898

[51] B. Sampson , M. K. Fagerhol , C. Sunderkötter , B. E. Golden , P. Richmond , N. Klein , I. Z. Kovar , J. H. Beattie , B. Wolska‐Kusnierz , Y. Saito , J. Roth , Lancet 2002, 360, 1742. 12480428 10.1016/S0140-6736(02)11683-7

[52] A. Lardner , J. Leukocyte Biol. 2001, 69, 522. 11310837

[53] B. D. Corbin , E. H. Seeley , A. Raab , J. Feldmann , M. R. Miller , V. J. Torres , K. L. Anderson , B. M. Dattilo , P. M. Dunman , R. Gerads , R. M. Caprioli , W. Nacken , W. J. Chazin , E. P. Skaar , Science 2008, 319, 962. 18276893 10.1126/science.1152449

[54] J. R. Stephan , F. Yu , R. M. Costello , B. S. Bleier , E. M. Nolan , J. Am. Chem. Soc. 2018, 140, 17444. 30380834 10.1021/jacs.8b06354 PMC6534964

[55] T. Vogl , N. Leukert , K. Barczyk , K. Strupat , J. Roth , Biochim. Biophys. Acta 2006, 11, 25. 10.1016/j.bbamcr.2006.08.028 17050004

[56] P. Bonaventura , G. Benedetti , F. Albarède , P. Miossec , Autoimmun Rev. 2015, 14, 277. 25462582 10.1016/j.autrev.2014.11.008

[57] J. M. Ehrchen , C. Sunderkötter , D. Foell , T. Vogl , J. Roth , J. Leukoc. Biol. 2009, 86, 557. 19451397 10.1189/jlb.1008647

[58] T. Vogl , M. Eisenblätter , T. Völler , S. Zenker , S. Hermann , P. van Lent , A. Faust , C. Geyer , B. Petersen , K. Roebrock , M. Schäfers , C. Bremer , J. Roth , Nat. Commun. 2014, 5, 4593. 25098555 10.1038/ncomms5593 PMC4143994

