# Supplementary material for: Alarming and Calming: Opposing Roles of S100A8/S100A9 Dimers and Tetramers on Monocytes
Source: Adv Sci (Weinh). 2022 Oct 30;9(36):2201505. doi: 10.1002/advs.202201505 (PMC9798971; doi:10.1002/advs.202201505)
Supplement: Supplementary file 1 — Supporting Information [file ADVS-9-2201505-s001.pdf]

ADVANCED SCIENCE
Open Access

# Supporting Information

for *Adv. Sci.*, DOI 10.1002/advs.202201505

Alarming and Calming: Opposing Roles of S100A8/S100A9 Dimers and Tetramers on Monocytes

*Antonella Russo, Hendrik Schürmann, Matthias Brandt, Katja Scholz, Anna Livia L. Matos, David Grill, Julian Revenstorff, Maximilian Rembrink, Meike von Wulffen, Lena Fischer-Riepe, Peter J. Hanley, Hans Häcker, Monika Prünster, Francisco Sánchez-Madrid, Sven Hermann, Luisa Klotz, Volker Gerke, Timo Betz, Thomas Vogl* and Johannes Roth*

**Alarming and calming: Opposing roles of S100A8/S100A9 dimers and tetramers on monocytes**

Antonella Russo[1,9], Hendrik Schürmann[2], Matthias Brandt[2], Katja Scholz[1], Anna Livia L. Matos[3,9], David Grill[3], Julian Revenstorff[1], Maximilian Rembrink[1], Meike von Wulffen[1], Lena Fischer-Riepe[1], Peter J. Hanley[4], Hans Häcker[5], Monika Prünster[6], Francisco Sánchez-Madrid[7,8], Sven Hermann[10], Luisa Klotz[11], Volker Gerke[3,9], Timo Betz[2,9,12], Thomas Vogl[1*§], and Johannes Roth[1,9§]

Supplementary Figure 1

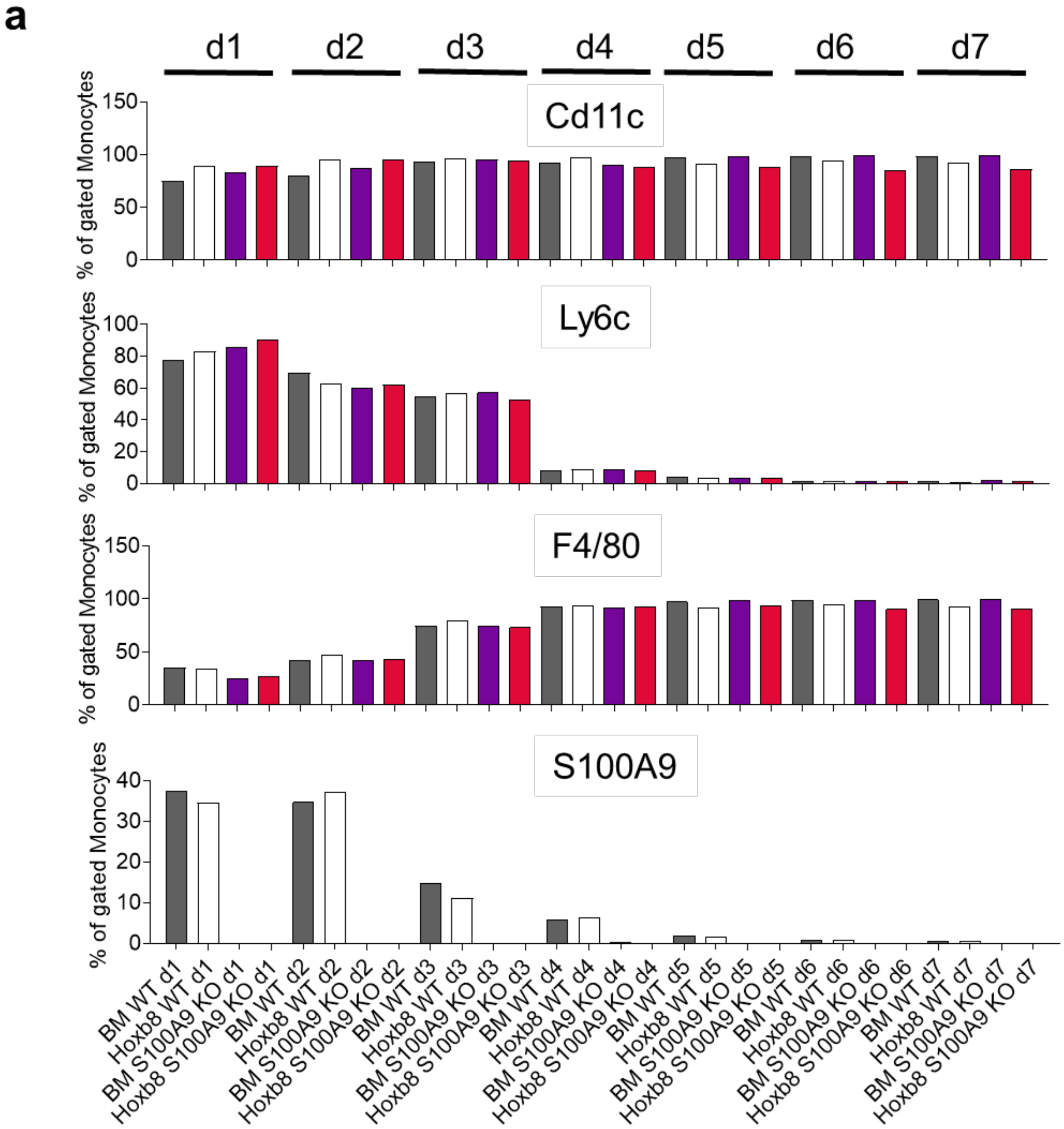


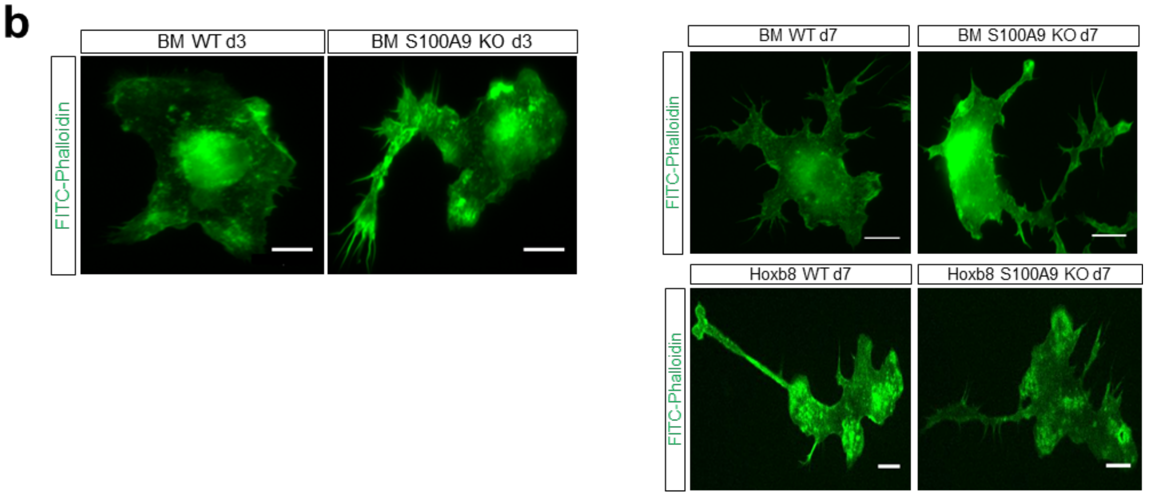

**Supp Fig. 1 Comparison of HoxB8 monocytes and BM derived monocytes.** Bone marrow of C57BL/6 WT and S100A9 knockout mice as well as Hoxb8 EGFP-Lifeact wild-type and S100A9 knockout cells were allowed to differentiate for up to seven days in the presence of M-CSF. **a**, Surface expression of Cd11b, Ly6c and F4/80 was analyzed for each day as well as the intracellular expression of S100A9 indicating no alterations in the differentiation of primary monocytes compared to HoxB8 monocytic cell lines. **b**, Morphological analysis of primary bone marrow derived monocytes (d3) and macrophages (d7). Cells were fixed and FITC-phalloidin labeled. Scale bar = 10µm.

# Supplementary Figure 2

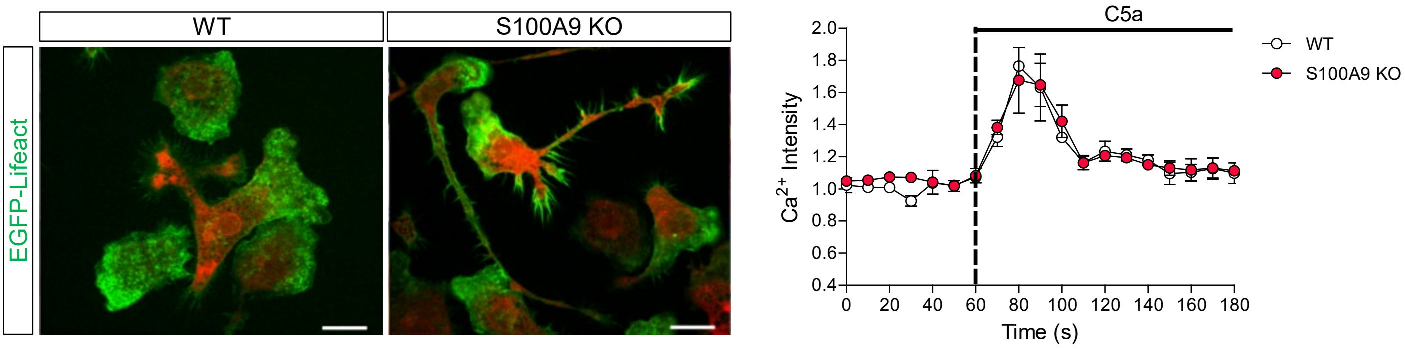


**Supp Fig. 2 Reactivity of Lifeact WT and Lifeact S100A9 KO cells to the complement C5a.** Screenshot of a live cell imaging time lap. Lifeact WT and Lifeact S100A9 KO monocytes stained for calcium with Cal590/AM (red) for 20 minutes. Images were taken every 10 seconds for 6 minutes. On the right panel the $Ca^{2+}$ intensity signal over time. Unpaired two-tailed *t*-test. Scale bar = 10 µm.

# Supplementary Figure 3

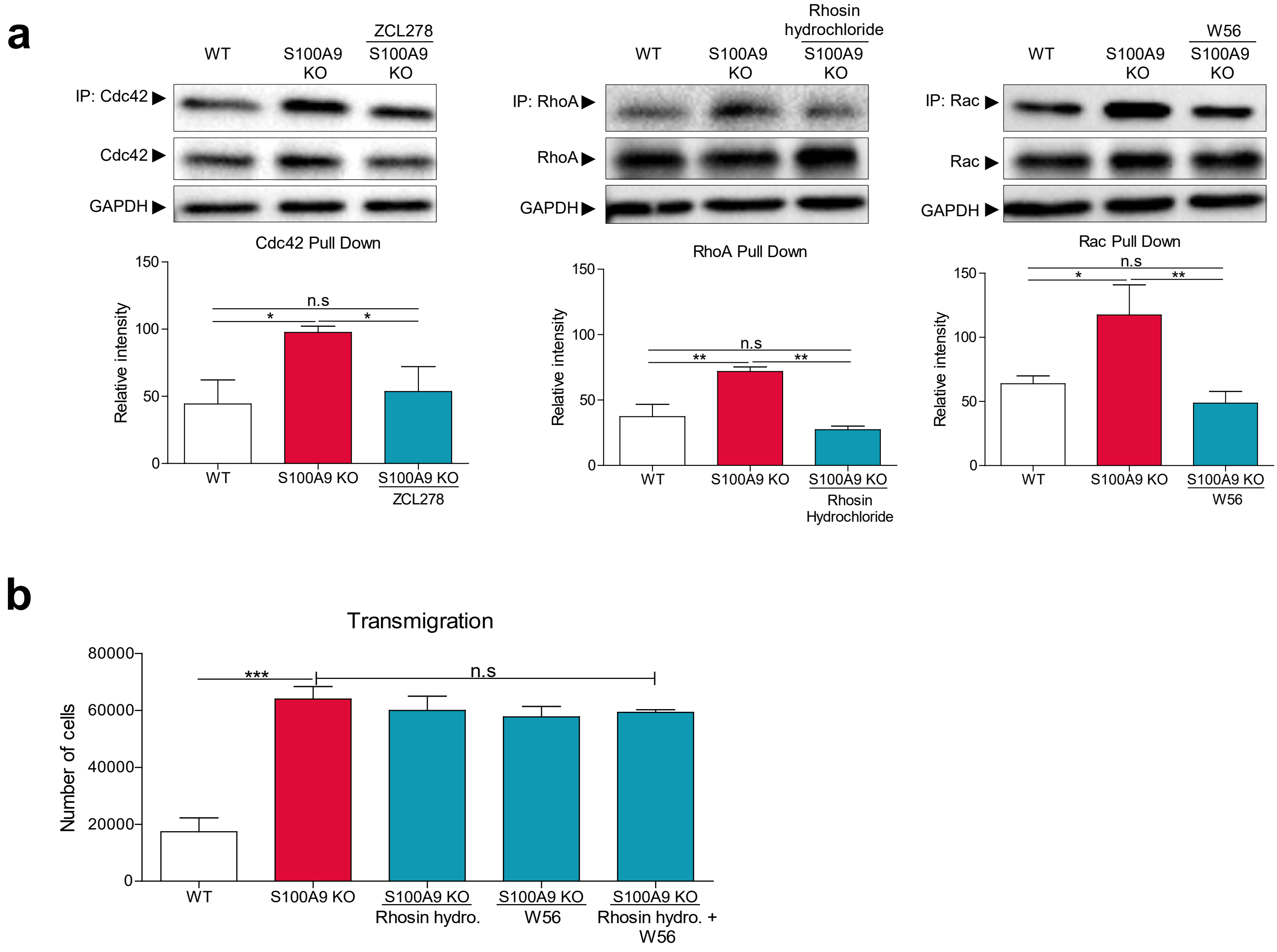


**Supp Fig. 3 Effect of S100A8/S100A9 on GTPases, migration.**

**a**, Immunoprecipitation assays of Cdc42, RhoA and Rac of Lifeact WT, Lifeact S100A9 KO and Lifeact S100A9 KO monocytes treated with ZCL278 (50 µM) or Rhosin hydrochloride (30 µM) or W56 (250 µM). **b**, Analysis of transmigration in a chemokine free boyden chamber assay of Lifeact WT, Lifeact S100A9 KO and Lifeact S100A9 KO cells treated with Rhosin hydrochloride (30µM), W56 (250µM) and with Rhosin hydrochloride and W56 simultaneously for 30 minutes.

Data are pooled from 3 independent experiments. For statistical analysis One-way ANOVA with Bonferroni post-test analysis was used n.s, not significant, * $P < 0.05$, ** $P < 0.01$, *** $P < 0.001$.

# Supplementary Figure 4

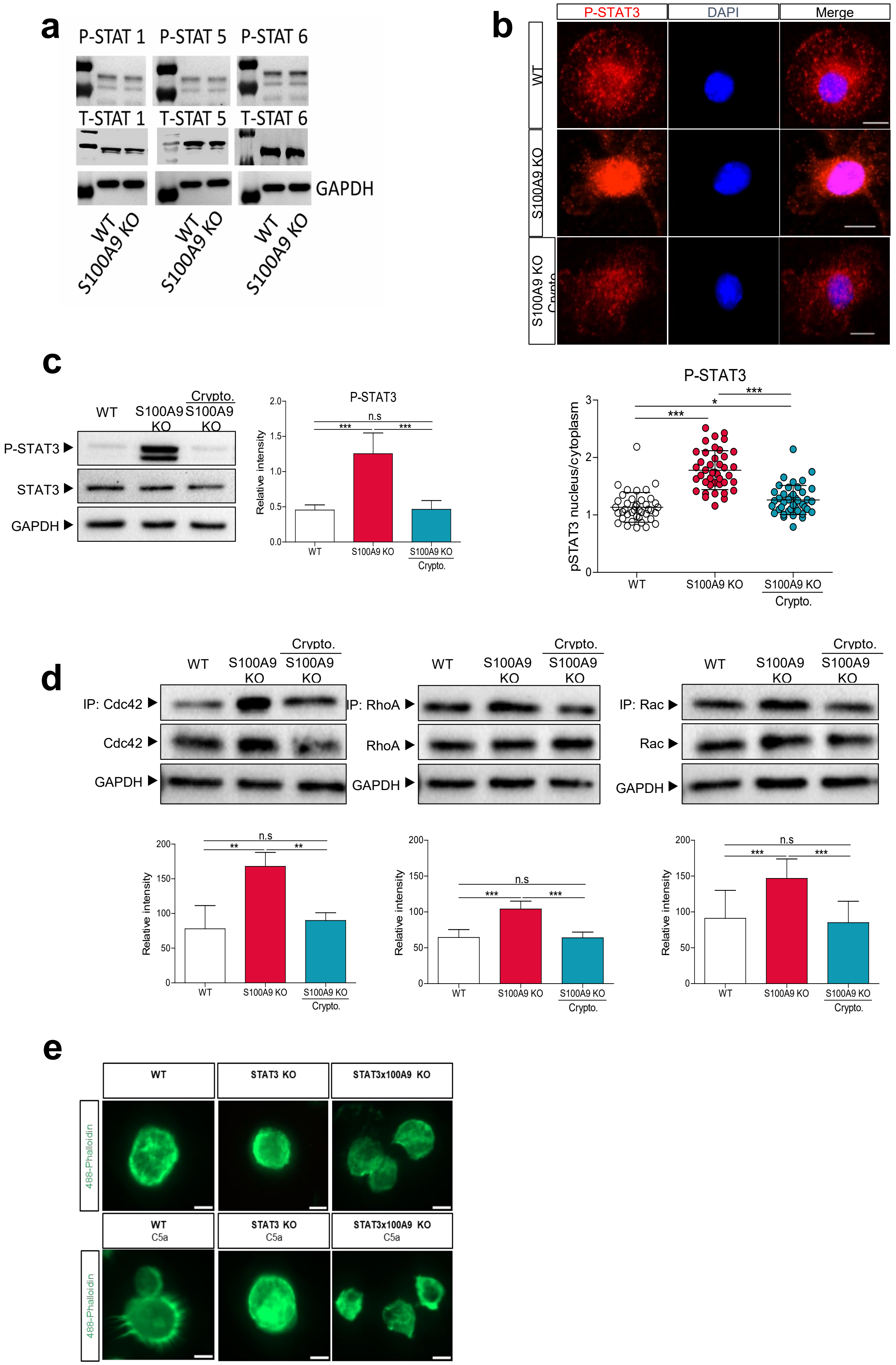

**Sup Fig. 4 STAT immunoblots, cryptotanshinone treatment of S100A9 KO monocytes and morphology of STAT3 KO and STAT3xS100A9 KO monocytes.**

**a**, Immunoblot analysis of P-STAT 1, 5 and 6 and total STAT 1, 5 and 6 of WT and S100A9 KO monocytes. **b**, Immunofluorescence staining of P-STAT3 in Lifeact WT (*n* = 41), Lifeact S100A9 KO (*n* = 41), and Lifeact S100A9 KO monocytes treated with Cryptotanshinone (Crypto., 5 µM, 30 minutes; *n* = 41). For statistical analysis the intensity of the P-STAT3 signal was measured via ImageJ in the nucleus and normalized to the signal in the cytoplasm. Scale bar = 10 µm. **c**, Immunoblot analysis of P-STAT3 and total STAT3 of Lifeact WT and Lifeact S100A9 KO monocytes as well as Lifeact S100A9 KO cells treated with Cryptotanshinone (Crypto., 5 µM, 30 minutes). **d**, Immunoprecipitation assays of Cdc42, RhoA and Rac. Lifeact WT and Lifeact S100A9 KO monocytes were left untreated or treated with Crypto. (5 µM for 30 minutes). **e**, Images of unstimulated WT-, STAT3 KO- and STAT3xS100A9 double KO monocytes (upper panel) or treated for 30 minutes with 20 nM of C5a (bottom panel). Data are pooled from 3 independent experiments. For statistical analysis One-way ANOVA with Bonferroni post-test analysis was used for all experiments. n.s, not significant, * $P < 0.05$, ** $P < 0.01$, *** $P < 0.001$.

# Supplementary Figure 5

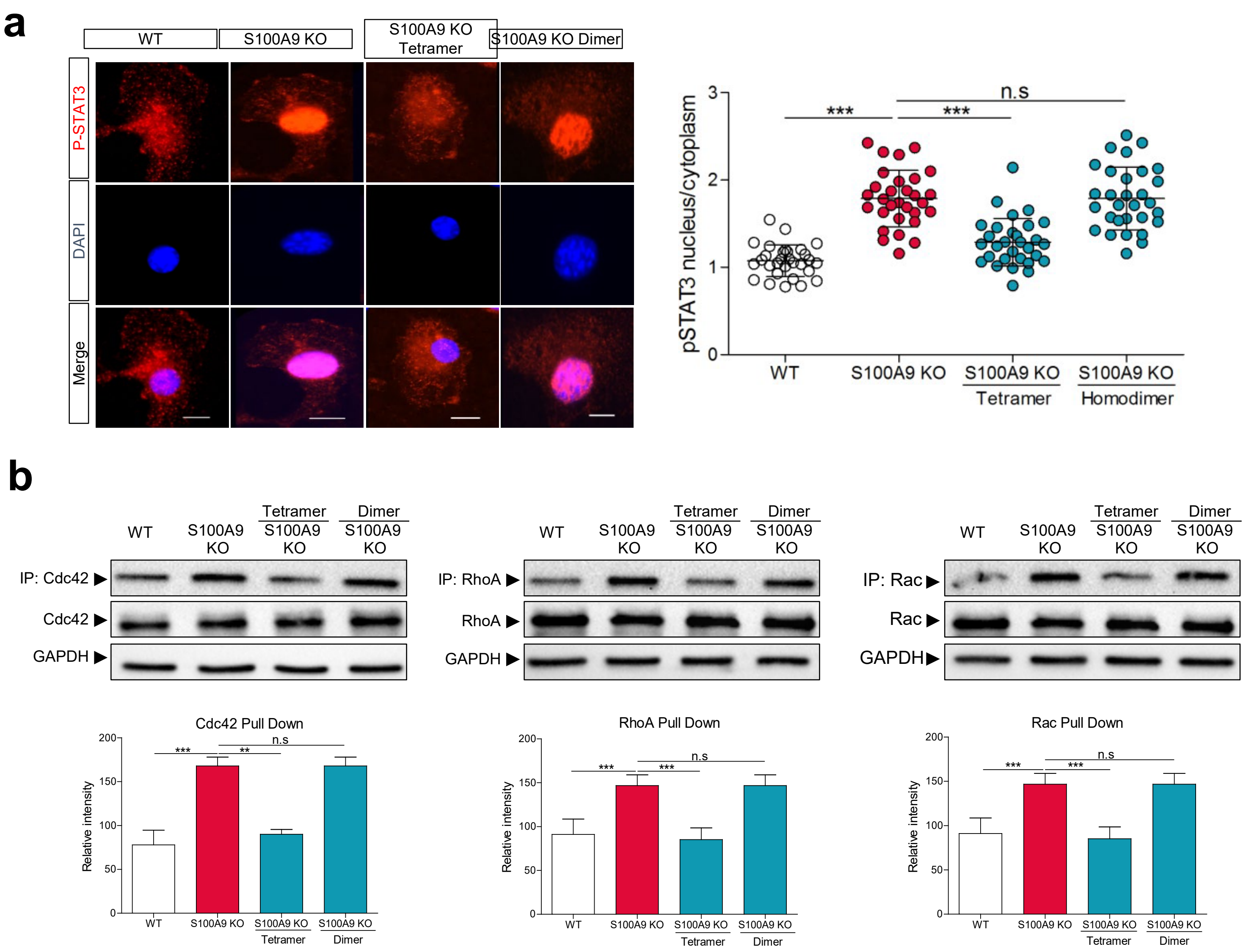


**Supp Fig. 5 Effect of the S100-tetramer and -homodimer on nuclear pSTAT3 localization and activity of the small Rho GTPases.**

**a**, Immunofluorescence staining of P-STAT3 in Lifeact WT (*n* = 29), Lifeact S100A9 KO (*n* = 30) and Lifeact S100A9 KO monocytes treated with S100A8/S100A9-tetramer (250 ng/ml; *n* = 30) or S100A8/S100A8-homodimer (250 ng/ml; *n* = 30). **b**, Immunoprecipitation assays of Cdc42, RhoA and Rac. Lifeact WT, Lifeact S100A9 KO as well as Lifeact S100A9 KO monocytes treated for 3 days with either S100A8/S100A9-tetramer (250 ng/ml) or S100A8/S100A8-homodimer (250 ng/ml). Data are pooled from 3 independent experiments. For statistical analysis One-way ANOVA with Bonferroni post-test analysis was used. n.s, not significant, ** $P < 0.01$, *** $P < 0.001$.

# Supplementary Figure 6

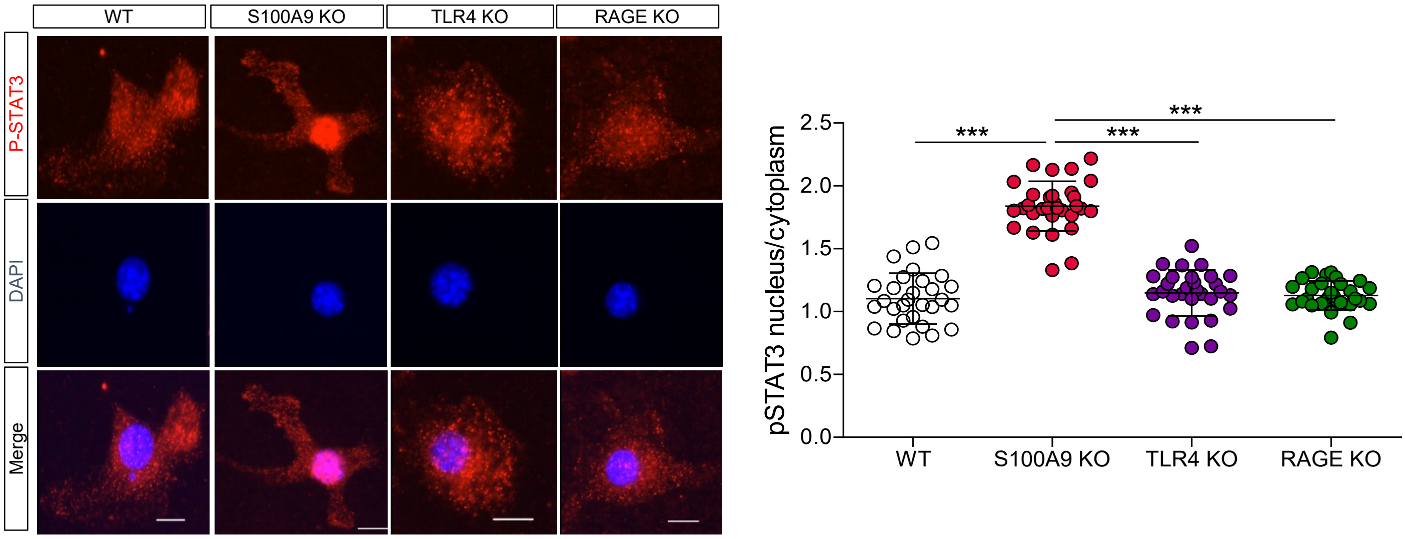


**Supp Fig. 6 STAT-3 localization in WT, S100A9 KO, TLR4 KO and RAGE KO monocytes.**

Immunofluorescence staining of P-STAT3 in Lifeact WT (*n* = 29), Lifeact S100A9 KO (*n* = 31), TLR4 KO (*n* = 30) and RAGE KO monocytes (*n* = 30).

Data are pooled from 3 independent experiments. For statistical analysis One-way ANOVA with Bonferroni post-test analysis was used for all experiments. n.s, not significant, ** $P < 0.01$, *** $P < 0.001$.

Supplementary Figure 7

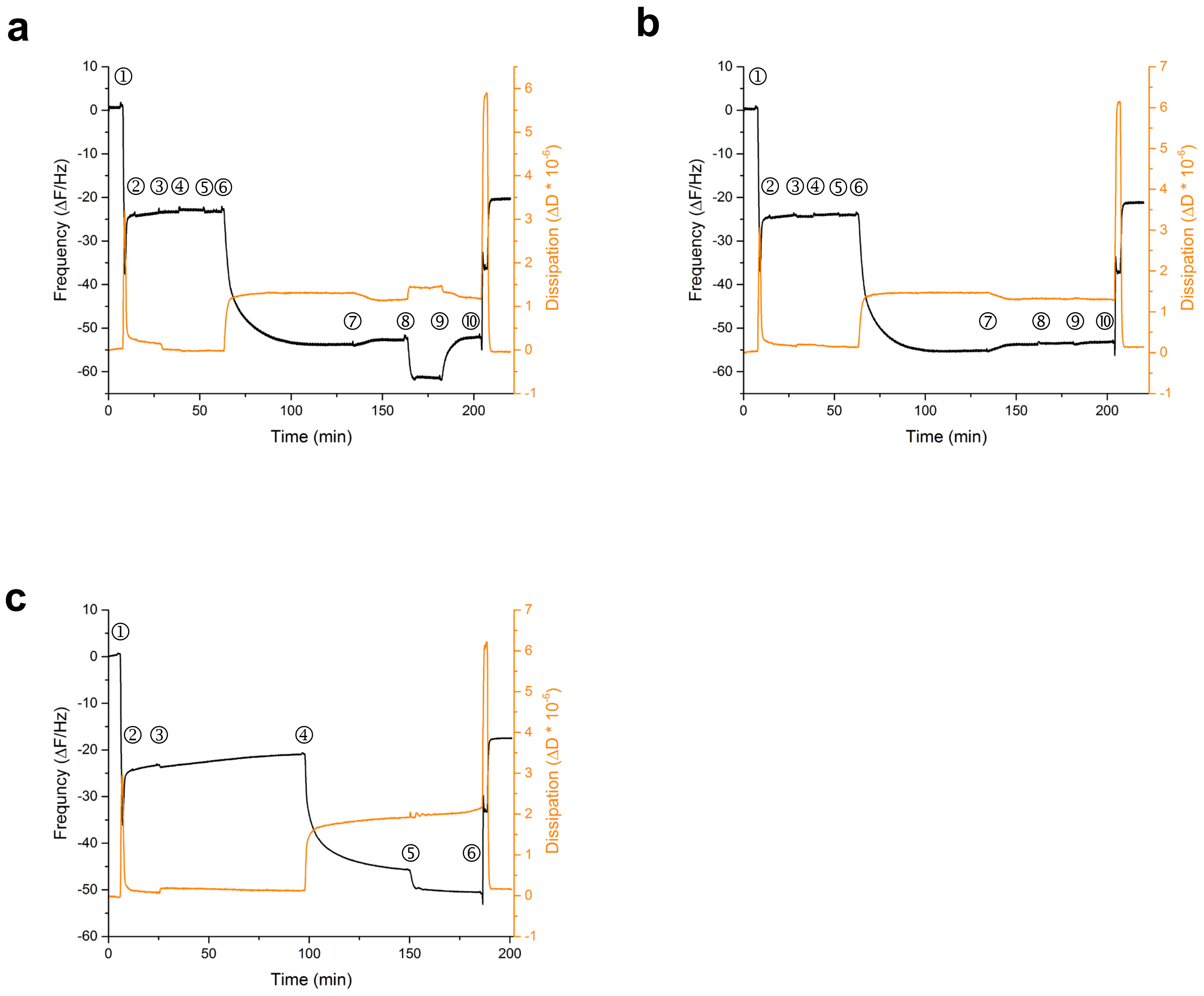


**Supp Fig. 7 Binding of S100-tetramer to CD69**

**a-c**, Quartz Crystal microbalance analysis, black lines show the resonance frequency and the orange lines the dissipation shifts of the quartz sensors during measurement over time. **a** and **b** spikes showing, (1) formation of the lipid bilayer. (2) change to citrate buffer. (3) change to HBS containing CaCl2. (4) Addition of S100A8/S100A9-tetramer (1 µg/ml) (**a**) or S100A8/S100A9-N70A-heterodimer (1 µg/ml) (**b**) in both conditions' adsorption does not add mass (no unspecific binding). (5) change to HBS containing CaCl2. (6) CD69 receptor (250 nM) adsorption onto the lipid bilayer. (7)

change to HBS containing CaCl2. (8) S100A8/S100A9-tetramer (1 µg/ml) adsorption adds mass without changing the dissipation, indicating specific binding and that the lipid film is not perturbed (**a**), S100A8/S100A9-N70A-heterodimer (1 µg/ml) adsorption does not add mass (no specific binding) (**b**). (9) change to HBS containing CaCl2. (10) recovery of the frequency baseline upon imidazole chelation indicates total desorption of the receptor from the lipid bilayer. **c**, (1) formation of the lipid bilayer. (2) change to citrate buffer. (3) change to HBS containing CaCl2. (4) CD69 receptor (250 nM) adsorption onto the lipid bilayer. (5) Galectin (1 µg/ml) adsorption adds mass without changing the dissipation, indicating that the lipid film is not perturbed. (6) the recovery of the frequency baseline upon imidazole chelation indicates total desorption of the receptor from the lipid bilayer.
